# OsVPS34-generated PI3P recruits GPA5/Rab5a to regulate post-Golgi glutelin trafficking in rice endosperm

**DOI:** 10.1093/plphys/kiag154

**Published:** 2026-03-18

**Authors:** Shanbin Xu, Mingqing Ma, Huanhuan Zhao, Aoni Zhou, Zi Li, Bo Li, Yuzhe He, Guiping Zhang, Hongping Cai, Chuanwei Gu, Ting Yu, Xue Yang, Lei Zhou, Yu Zhang, Erchao Duan, Xuan Teng, Xi Liu, Shijia Liu, Yunlu Tian, Ling Jiang, Yulong Ren, Yihua Wang, Hui Dong, Jianmin Wan

**Affiliations:** State Key Laboratory of Crop Genetics & Germplasm Enhancement and Utilization, Zhongshan Biological Breeding Laboratory, Jiangsu Nanjing Rice Germplasm Resources National Field Observation and Research Station, Nanjing Agricultural University, Nanjing 210095, China; State Key Laboratory of Crop Genetics & Germplasm Enhancement and Utilization, Zhongshan Biological Breeding Laboratory, Jiangsu Nanjing Rice Germplasm Resources National Field Observation and Research Station, Nanjing Agricultural University, Nanjing 210095, China; State Key Laboratory of Crop Genetics & Germplasm Enhancement and Utilization, Zhongshan Biological Breeding Laboratory, Jiangsu Nanjing Rice Germplasm Resources National Field Observation and Research Station, Nanjing Agricultural University, Nanjing 210095, China; State Key Laboratory of Crop Genetics & Germplasm Enhancement and Utilization, Zhongshan Biological Breeding Laboratory, Jiangsu Nanjing Rice Germplasm Resources National Field Observation and Research Station, Nanjing Agricultural University, Nanjing 210095, China; State Key Laboratory of Crop Genetics & Germplasm Enhancement and Utilization, Zhongshan Biological Breeding Laboratory, Jiangsu Nanjing Rice Germplasm Resources National Field Observation and Research Station, Nanjing Agricultural University, Nanjing 210095, China; State Key Laboratory of Crop Genetics & Germplasm Enhancement and Utilization, Zhongshan Biological Breeding Laboratory, Jiangsu Nanjing Rice Germplasm Resources National Field Observation and Research Station, Nanjing Agricultural University, Nanjing 210095, China; State Key Laboratory of Crop Genetics & Germplasm Enhancement and Utilization, Zhongshan Biological Breeding Laboratory, Jiangsu Nanjing Rice Germplasm Resources National Field Observation and Research Station, Nanjing Agricultural University, Nanjing 210095, China; State Key Laboratory of Crop Genetics & Germplasm Enhancement and Utilization, Zhongshan Biological Breeding Laboratory, Jiangsu Nanjing Rice Germplasm Resources National Field Observation and Research Station, Nanjing Agricultural University, Nanjing 210095, China; State Key Laboratory of Crop Genetics & Germplasm Enhancement and Utilization, Zhongshan Biological Breeding Laboratory, Jiangsu Nanjing Rice Germplasm Resources National Field Observation and Research Station, Nanjing Agricultural University, Nanjing 210095, China; State Key Laboratory of Crop Genetics & Germplasm Enhancement and Utilization, Zhongshan Biological Breeding Laboratory, Jiangsu Nanjing Rice Germplasm Resources National Field Observation and Research Station, Nanjing Agricultural University, Nanjing 210095, China; State Key Laboratory of Crop Genetics & Germplasm Enhancement and Utilization, Zhongshan Biological Breeding Laboratory, Jiangsu Nanjing Rice Germplasm Resources National Field Observation and Research Station, Nanjing Agricultural University, Nanjing 210095, China; State Key Laboratory of Crop Genetics & Germplasm Enhancement and Utilization, Zhongshan Biological Breeding Laboratory, Jiangsu Nanjing Rice Germplasm Resources National Field Observation and Research Station, Nanjing Agricultural University, Nanjing 210095, China; State Key Laboratory of Crop Genetics & Germplasm Enhancement and Utilization, Zhongshan Biological Breeding Laboratory, Jiangsu Nanjing Rice Germplasm Resources National Field Observation and Research Station, Nanjing Agricultural University, Nanjing 210095, China; State Key Laboratory of Crop Genetics & Germplasm Enhancement and Utilization, Zhongshan Biological Breeding Laboratory, Jiangsu Nanjing Rice Germplasm Resources National Field Observation and Research Station, Nanjing Agricultural University, Nanjing 210095, China; State Key Laboratory of Crop Genetics & Germplasm Enhancement and Utilization, Zhongshan Biological Breeding Laboratory, Jiangsu Nanjing Rice Germplasm Resources National Field Observation and Research Station, Nanjing Agricultural University, Nanjing 210095, China; State Key Laboratory of Crop Genetics & Germplasm Enhancement and Utilization, Zhongshan Biological Breeding Laboratory, Jiangsu Nanjing Rice Germplasm Resources National Field Observation and Research Station, Nanjing Agricultural University, Nanjing 210095, China; State Key Laboratory of Crop Genetics & Germplasm Enhancement and Utilization, Zhongshan Biological Breeding Laboratory, Jiangsu Nanjing Rice Germplasm Resources National Field Observation and Research Station, Nanjing Agricultural University, Nanjing 210095, China; State Key Laboratory of Crop Genetics & Germplasm Enhancement and Utilization, Zhongshan Biological Breeding Laboratory, Jiangsu Nanjing Rice Germplasm Resources National Field Observation and Research Station, Nanjing Agricultural University, Nanjing 210095, China; State Key Laboratory of Crop Genetics & Germplasm Enhancement and Utilization, Zhongshan Biological Breeding Laboratory, Jiangsu Nanjing Rice Germplasm Resources National Field Observation and Research Station, Nanjing Agricultural University, Nanjing 210095, China; State Key Laboratory of Crop Genetics & Germplasm Enhancement and Utilization, Zhongshan Biological Breeding Laboratory, Jiangsu Nanjing Rice Germplasm Resources National Field Observation and Research Station, Nanjing Agricultural University, Nanjing 210095, China; State Key Laboratory of Crop Gene Resources and Breeding, National Key Facility for Crop Gene Resources and Genetic Improvement, Institute of Crop Sciences, Chinese Academy of Agricultural Sciences, Beijing 100081, China; State Key Laboratory of Crop Genetics & Germplasm Enhancement and Utilization, Zhongshan Biological Breeding Laboratory, Jiangsu Nanjing Rice Germplasm Resources National Field Observation and Research Station, Nanjing Agricultural University, Nanjing 210095, China; State Key Laboratory of Crop Genetics & Germplasm Enhancement and Utilization, Zhongshan Biological Breeding Laboratory, Jiangsu Nanjing Rice Germplasm Resources National Field Observation and Research Station, Nanjing Agricultural University, Nanjing 210095, China; State Key Laboratory of Crop Genetics & Germplasm Enhancement and Utilization, Zhongshan Biological Breeding Laboratory, Jiangsu Nanjing Rice Germplasm Resources National Field Observation and Research Station, Nanjing Agricultural University, Nanjing 210095, China; State Key Laboratory of Crop Gene Resources and Breeding, National Key Facility for Crop Gene Resources and Genetic Improvement, Institute of Crop Sciences, Chinese Academy of Agricultural Sciences, Beijing 100081, China

## Abstract

Seed storage proteins (SSPs) are stored in protein storage vacuoles (PSVs) within plant endosperm cells. In rice, glutelins undergo post-Golgi trafficking via dense vesicles (DVs) to protein body II (PBII). Phosphatidylinositol 3-phosphate (PI3P) regulates endosomal, autophagic, and vacuolar trafficking, yet its role in glutelin transport remains unclear. Here, we characterized the *glutelin precursor accumulation14* (*gpa14*) mutant, which exhibits over-accumulation of 57-kDa glutelin precursors and floury, shrunken endosperm. Map-based cloning identified a single adenine insertion in *Vacuolar Protein Sorting 34* (*OsVPS34*), resulting in a putative truncated protein lacking the PI3Ka and PI3_PI4_kinase domains. *OsVPS34* encodes phosphatidylinositol 3-kinase (PI3K), which interacts with other subunits of the PI3K complex to regulate the production of PI3P. PI3P was enriched in the trans-Golgi network (TGN) and prevacuolar compartment (PVC), co-localized with Rab5a and GPA5, and was detected in DVs and PBIIs. In *gpa14*, PI3P levels were reduced, leading to mis-localization and decreased membrane association of Rab5a and GPA5, key regulators of glutelin trafficking. Our findings demonstrate that OsVPS34 is essential for synthesis of PI3P, which plays a crucial role in recruiting GPA5 and Rab5a to DVs for glutelin post-Golgi trafficking in rice endosperm.

## Teaser

PI3K-dependent PI3P production recruits trafficking regulators Rab5a and GPA5 to dense vesicles and ensures efficient post-Golgi transport of glutelins in rice endosperm.

## Introduction

Rice (*Oryza sativa* L.), a globally important staple crop, relies on seed storage proteins (SSPs) as a crucial nutrient component, second only to starch, comprising approximately 8% to 10% of the dry mass in mature caryopsis (Muthayya et al. 2014; Long et al. 2023). Based on their solubility, SSPs are categorized into glutelin (60% to 80% of total SSPs), prolamin (20% to 30%), globulin (2% to 8%), and albumin (approximately 5%) (Pan and Reeck 1988; Shorrosh et al. 1992; Yang et al. 2019; He et al. 2021). Glutelins are synthesized as 57-kDa precursors, which are subsequently cleaved into 22- to 23-kDa basic subunits and 37- to 39-kDa acidic subunits before being stored in protein body II (PBII) (Ren et al. 2022). In rice endosperm, glutelins undergo a complex trafficking process: they are initially synthesized in the endoplasmic reticulum (ER), processed in the Golgi apparatus, and then sorted from the trans-Golgi network (TGN) via dense vesicles (DVs, uniform vesicles approximately 100 to 200 nm in diameter) or storage prevacuolar compartments (sPVCs) to PBⅡ (Wang et al. 2010; Shen et al. 2011; Liu et al. 2013; Pan et al. 2021; Zhang et al. 2025). Within PBII, vacuolar processing enzymes cleave the 57-kDa precursors into their mature subunits, ensuring proper deposition and storage (Wang et al. 2009).

57H mutants characterized by the overaccumulation of the 57-kDa glutelin precursor have been demonstrated to be ideal genetic materials for identifying regulators involved in glutelin trafficking (Takemoto et al. 2002; Ueda et al. 2010; Ren et al. 2022). Endosperm storage protein 2 (ESP2)/PDI, a protein disulfide isomerase, facilitates glutelin folding by forming intramolecular disulfide bonds between glutelin subunits and enables ER export (Takemoto et al. 2002). GPA4/GOT1B (for the Golgi transport 1B), GPA12/(SEC13 homolog A) Sec13a, and GPA13/(Secretion-associated Ras super family 1c) Sar1c have been reported to regulate glutelin export from the ER (Wang et al. 2016, 2024; Bao et al. 2023). Several protein regulators are specifically involved in DV-mediated post-Golgi trafficking, including GPA1/GLUP4/Rab5a, GPA2/GLUP6/VPS9a, GPA3, GPA5, and GPA7. GPA3 recruits GPA2/GLUP6/VPS9a, which functions as a guanine exchange factor (GEF) for GPA1/GLUP2/Rab5a. These proteins form functional complexes to regulate post Golgi trafficking (Ren et al. 2014). *GPA5* encodes a protein with an N-terminal PX domain and a C-terminal coiled-coil (CC) domain and functions as the effector of Rab5a to regulate the fusion of DV to PSV (Wang et al. 2010; Liu et al. 2013; Ren et al. 2014). Additionally, the GPA7/MONENSIN SENSITIVITY1-CALCIUM CAFFEINE ZINC SENSITIVITY1 (MON1-CCZ1) complex acts as Rab7-GEF and is involved in post-Golgi trafficking via the sPVC-mediated pathway (Pan et al. 2021). Although these studies have outlined our basic understanding of DV-mediated glutelin sorting in rice, further identification of additional regulator is necessary to deepen our knowledge of glutelin trafficking.

The phosphatidylinositol 3-kinases (PI3Ks), initially identified in yeast as Vps34p (Schu et al. 1993), are lipid kinases that catalyze the production of phosphoinositide. PI3K in animals are classified into 3 types, PI3KⅠ, PI3KⅡ, and PI3KⅢ, each synthesizing a distinct phosphoinositide: phosphatidylinositol 3,4,5-trisphosphate (PI(3,4,5)P_3_), phosphatidylinositol 3,4-bisphosphate (PI(3,4)P_2_), and phosphatidylinositol 3-phosphate (PI3P), respectively. Among these, only PI3KⅢ is evolutionarily conserved across animals, plants, yeast, and algae, which exclusively produces PI3P (Liu et al. 2020). In plants, the PI3K complex comprises 4 subunits, 1 kinase vacuolar protein sorting 34 (VPS34) and 3 accessory proteins kinase vacuolar protein sorting 15 (VPS15), kinase vacuolar protein sorting 30 (VPS30) (also known as Beclin 1 or ATG6), along with either kinase vacuolar protein sorting 38 (VPS38) or autophagy-related protein 14 (ATG14). Typically, PI3K complex I (PI3KCI), consisting of VPS34, VPS15, VPS30/ATG6, and ATG14, is primarily involved in autophagy. In contrast, PI3K complex II (PI3KCII), comprising VPS34, VPS15, VPS30/ATG6, and VPS38, functions in the endosomal and vacuolar protein sorting pathways (Itakura et al. 2008; Lee et al. 2018). Notably, PI3KCII is also required for macro-autophagy (one type of autophagic process) induction under nutrient-stressed conditions (Tasnin et al. 2022). In both PI3K complexes, the core subunits, VPS34-VPS15-VPS30/ATG6, are essential for the catalytic activity of PI3K. Both PI3P and PI3K have been reported to regulate normal plant growth, symbiosis, and responses to abiotic and biotic stresses (Lee et al. 2008b; Noack and Jaillais 2017). These regulatory functions have been demonstrated through the disruption of the PI3K complex function through antisense constructs targeting core subunits, tissue-specific expressing PI3P-binding proteins (such as FYVE domain-containing proteins such like Fab1, YOTB, Vac1, and EEA1), or chemical inhibition with wortmannin/LY294002 (Joo et al. 2005; Lee et al. 2008a). However, the lethality of homozygous mutations in *VPS34*, *VPS30*/*ATG6*, and *VPS15* presents a major obstacle to fully understanding the functions of PI3K in agronomical trait formation in crops (Fujiki et al. 2007; Qin et al. 2007; Harrison-Lowe and Olsen 2008; Wang et al. 2012).

PI3P is a crucial endomembrane signaling molecule that regulates endosomal, autophagic, and vacuolar/lysosomal trafficking by recruiting effector proteins (Nascimbeni et al. 2017; Wallroth and Haucke 2018). Most of these effectors contain either Fab1, YGL023, Vps27, EEA1, (FYVE) or Phox homology (PX) domains (Noack and Jaillais 2017; Di Sansebastiano et al. 2018; Marshall and Vierstra 2018). PI3P is synthesized by PI3K, which phosphorylates the third position of the phosphatidylinositol (PI) head group. In macro-autophagy, PI3P is enriched in the phagophore membrane, where it facilitates autophagosome expansion and formation by recruiting proteins such as SH3 domain-containing protein 2 (SH3P2), which associates with ATG8-PE (Zhuang et al. 2013). Beyond its role in autophagy, PI3P is also essential for endosomal sorting and vacuolar transport. In animals and yeast, PI3P recruits FYVE domain protein such as Hrs/Vps27p, a key component of the endosomal sorting complexes required for transport (ESCRT) machinery. Hrs/Vps27p interacts with tumor susceptibility gene 101 (Tsg101) to mediate ESCRT-I recruitment to early endosomes (Bache et al. 2003). In plants, FREE1/FYVE1, a PI3P effector, is an integral component of the ESCRT machinery, playing a crucial role in multivesicular body (MVB) biosynthesis and MVB-mediated membrane protein sorting through interactions with ESCRT-I subunit Vps23 (Gao et al. 2014; Kolb et al. 2015; Belda-Palazon et al. 2016). During autophagosome maturation, PI3P-associated ATG8-coated autophagosomes are transported to the vacuole via PI3P effectors such as FYVE domain protein required for endosomal sorting 1 (FREE1)/FYVE1. Additionally, FYVE4, another PI3P effector, interacts with ESCRT-III subunit SNF7 to regulate intraluminal vesicle (ILV) formation within MVBs (Liu et al. 2021). PI3P also participates in the vesicle trafficking and vacuolar sorting pathways by recruiting PI3P-binding proteins, such as ADL6 (PH), NaPCCP (C2), and EpsnR2 (ENTH) (Lee et al. 2002, 2007, 2009). Moreover, in rice, PI3P plays a key role in glutelin trafficking by specifically binding to GPA5 (Ren et al. 2020). However, the precise subcellular localization of PI3P enrichment and how PI3P functions in the post-Golgi trafficking of glutelin remain poorly understood.

In this study, we isolated a mutant from a tissue culture–derived mutagenesis library, named *glutelin precursor accumulation 14* (*gpa14*). Homozygous *gpa14* mutant is seedling lethal and exhibits floury, shrunken endosperm, an over-accumulated 57-kDa glutelin precursor, incompletely filled PBII, and abnormal paramural body (PMB) structures in grains. *GPA14* encodes phosphatidylinositol 3-kinase and was named *Vacuolar protein sorting 34* (*OsVPS34*). We demonstrate that OsVPS34 as a key subunit of the PI3K complex is essential for PI3P production. Using immunogold electron microscopy, PI3P was detected in DV and PBⅡ. Additionally, we found that the PI3P plays a critical role in maintaining the localization of GPA1/Rab5a and GPA5 to the PVC. Overall, our findings characterize the function of OsVPS34 in PI3P production, delineate the assembly of the PI3K complex, and elucidate how PI3P regulates glutelin post-Golgi trafficking from DVs to PBIIs as a signal phosphoinositide in rice.

## Results

### The *gpa14* mutant exhibits over-accumulated 57-kDa glutelin precursor

**Figure 1:**
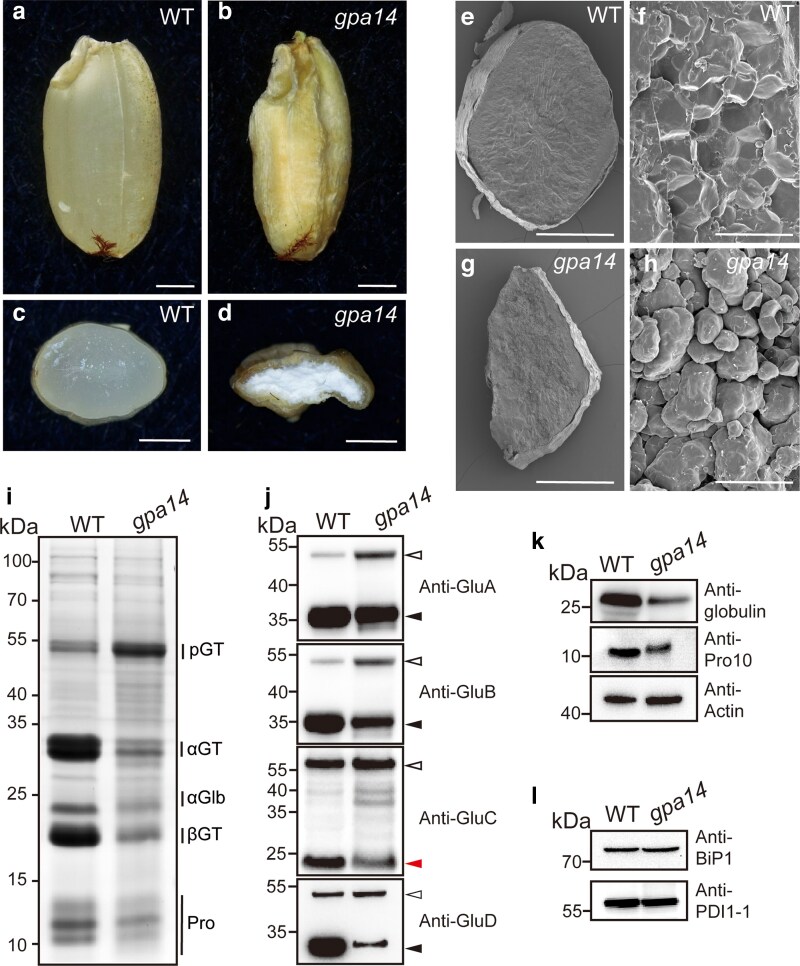
Phenotypic characterization of the *gpa14* mutant. **a–b)** Mature grains of wild type (WT) (a) and *gpa14* (b). Scale bars represent 2 mm. **c–d)** Transverse sections of mature grains from WT (c) and *gpa14* (d). Scale bars represent 2 mm. **e–h)** Scanning electron microscopy (SEM) images of transverse sections of mature grains in WT (e, g) and *gpa14* (f, h). Scale bars: 1 mm for panels e and f; 20 µm for panels g and h. **i)** SDS-PAGE analysis of total proteins from mature seeds of WT and *gpa14*, visualized by CBB staining. Abbreviations: pGT, unprocessed glutelin precursors; αGT, mature acidic subunits of glutelins; αGlb, 26 kDa α-globulin; βGT, mature basic subunits of glutelins; Pro, prolamins. **j)** Immunoblot analysis of storage proteins with anti-glutelin subfamily-specific antibodies (GluA/B/C/D). White, black, and red arrows indicate the 57-kDa glutelin precursors, glutelin acidic subunits and glutelin basic subunits, respectively. **k–l)** Immunoblot analysis of storage proteins with anti-α-globulin and anti-Pro10 antibodies (k) and anti-molecular chaperone (BiP1 and PDI1-1) antibodies (l). Actin served as a loading control in panels (j–l).

The *gpa14* mutant was isolated from a tissue culture–derived mutagenesis library *japonica* cv. Ningjing 4. Due to seedling lethality, homozygous *gpa14* seeds were obtained from the segregation of heterozygous individuals. These homozygous seeds exhibited a floury, shrunken endosperm (Fig. 1a–d). Scanning electron microscopy (SEM) of mature seeds revealed that the starch granules in *gpa14* were spherical and loosely arranged, in contrast to the tightly packed, polyhedral starch granules observed in the wild type (WT) (Fig. 1e–h). SDS-PAGE analysis of mature seeds from WT and *gpa14* showed a marked over-accumulation of 57-kDa glutelin precursors in the mutant, along with reductions in glutelin acidic subunits, glutelin basic subunits, 26-kDa α-globulin, and prolamins (Fig. 1i). The accumulation of glutelin precursors in the *gpa14* was further confirmed by immunoblotting with specific antibodies against GluA, GluB, GluC, and GluD (Fig. 1j). In addition, both SDS-PAGE and immunoblot analyses demonstrated decreased levels of globulin and prolamin in *gpa14* compared with WT (Fig. 1i, k). As previously reported, an increased abundance of molecular chaperones such as BINDING PROTEIN1 (BiP1) and PDI1-1 typically often suggests the defective ER exit of SSPs (Takemoto et al. 2002; Yasuda et al. 2009; Wang et al. 2016, 2024; Bao et al. 2023). However, we observed no detectable differences in the abundance of BiP1 and PDI1-1 between WT and *gpa14*, suggesting that overaccumulation of the 57-kDa glutelin precursors in *gpa14* is not due to impaired ER exit (Fig. 1l). Therefore, the *gpa14* mutation likely affects the trafficking of proglutelins.

### The *gpa14* mutant exhibits disrupted sorting of storage proteins

**Figure 2:**
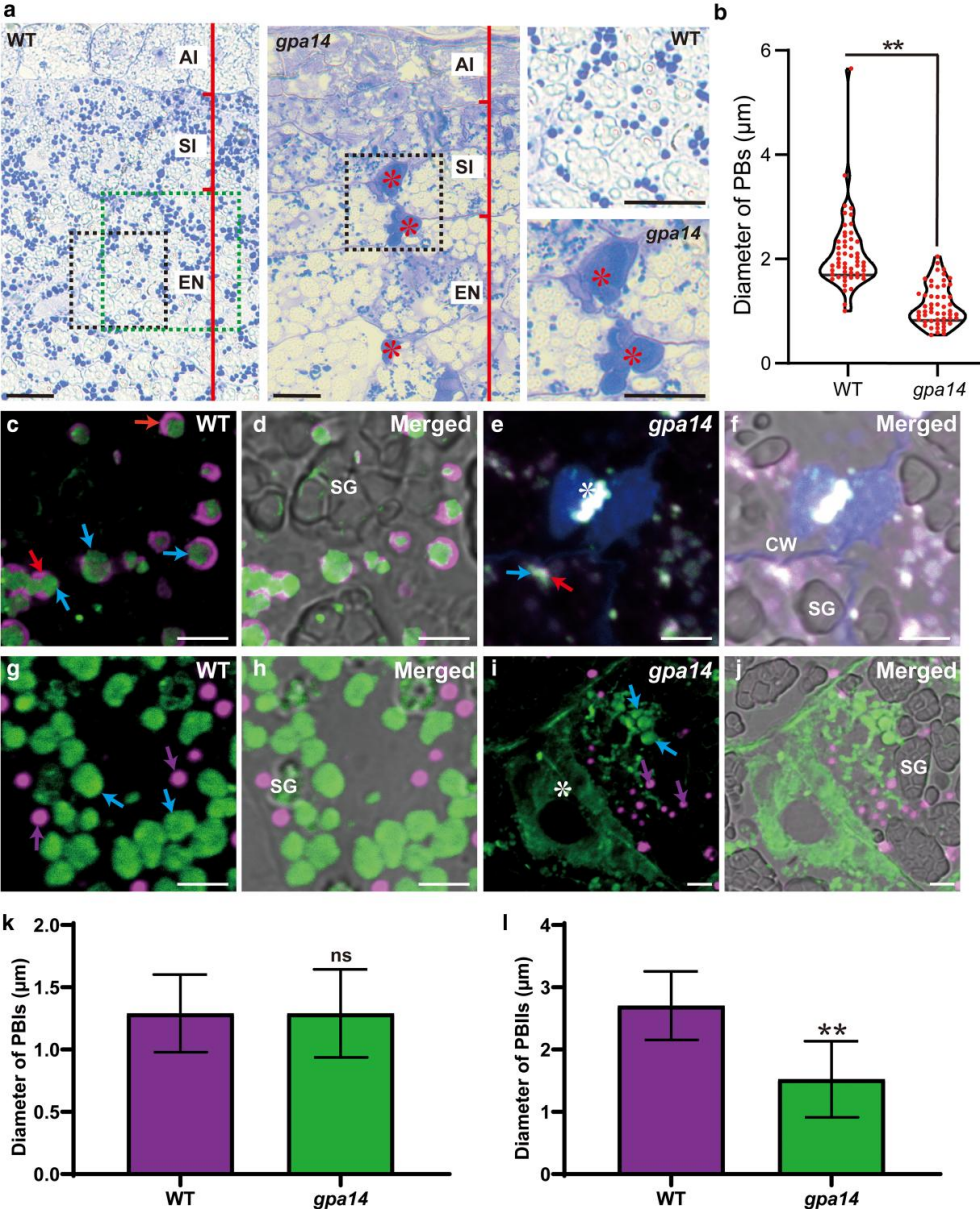
Aberrant sorting of glutelin into PMBs in the *gpa14* mutant. **a)** Light microscopy observation of CBB-stained sections from WT and *gpa14* at 12 DAF seeds. The right side of this panel is magnified areas, which displays the black dashed boxes within the images on the left and middle of this panel. Red lines demarcate 3 endosperm cell types: Al, aleurone layer; EN, starch endosperm; Sl, sub-aleurone layer. Asterisks denote PMB structures. Scale bars = 20 µm. **b)** Quantification of PB diameter at 12 DAF in WT and *gpa14* mutant. Within the violin plots, the horizontal black lines indicate the medians, the shape shows the data distribution density, and the red dots represent individual measurements. Asterisks indicate statistically significant difference between WT and *gpa14*, determined by Student *t* tests. Data represent mean ± SD (*n* > 50; **P < 0.01). **c–f)** Immunofluorescence microscopy of glutelins (green) and α-globulin (magenta) in sub-aleurone layer cells of 12 DAF endosperm in WT and the *gpa14* mutant. Blue arrows indicate glutelins, red arrows indicate α-globulin, and white asterisk marks PMB structures. Abbreviations: CW, cell wall; SG, starch granule. Scale bars = 5 µm. **g–j)** Immunofluorescence microscopy of glutelin (green) and prolamins (magenta) in sub-aleurone layer cells of 12 DAF endosperm in WT and the *gpa14* mutant. Blue arrows indicate PBIIs, magenta arrows denote PBIs, and white asterisks mark the PMB structures. Scale bars = 5 µm. **k–l)** Quantification of PBI (k) and PBII (l) diameters. Asterisks indicate statistically significant difference between WT and *gpa14*, determined by Student *t* tests. Data represent mean ± SD (*n* > 50; ns, not significant, *P* > 0.05; *P < 0.05; **P < 0.01).

To investigate the cytological basis of abnormal glutelin precursor accumulation in the *gpa14* mutant, we prepared semi-thin sections of developing endosperm at 12 d after flowering (DAF) for both WT and *gpa14*. First, we performed Coomassie Brilliant Blue (CBB) staining to visualize proteins in the semi-thin sections. SSPs were predominantly deposited in the sub-aleurone cells of both WT and *gpa14* endosperm (Fig. 2a). In WT, SSPs were detected in round-shaped PBI and irregularly shaped PBII structures. However, in the *gpa14* mutant, SSPs were missorted to the extracellular space, forming aberrant PMB structures (Fig. 2a). We then measured the PB sizes and found that those in WT were significantly larger than those in the *gpa14* mutant (Fig. 2b).

To further investigate the subcellular distribution of SSPs in WT and the *gpa14* mutant, we performed double immunofluorescence labeling on developing endosperm at 12 DAF using antibodies specific to glutelins, α-globulin, and prolamins. Additionally, the cell wall was stained by calcofluor white. In WT, glutelins (green fluorescence) and α-globulin (magenta fluorescence) accumulated in the PBⅡs (Fig. 2c–d, g–h; Fig. S1a–b), with α-globulin specifically localized to the periphery of PBIIs (Fig. 2c–d). Prolamins (magenta fluorescence) were exclusively sequestered in PBIs (Fig. 2g–j; Fig. S1). However, in the *gpa14* mutant, some glutelins and α-globulin abnormally accumulated around the cell wall, forming PMB structures (Fig. 2e–f, i–j; Fig. S1c–d). Moreover, glutelins and α-globulin were mislocalized together (Fig. 2e–f), whereas prolamins were not apparently affected (Fig. 2i–j; Fig. S1c–d). Statistical analysis revealed no significant difference in PBI size between the WT and *gpa14* (Fig. 2k), In contrast, PBⅡ sizes were significantly smaller in the *gpa14* mutant compared with WT (Fig. 2l). These observations are consistent with the CBB staining results, indicating that the reduction in PB size is primarily due to smaller PBIIs rather than PBIs.

### The *gpa14* mutant results in abnormal sorting after DV budding

**Figure 3:**
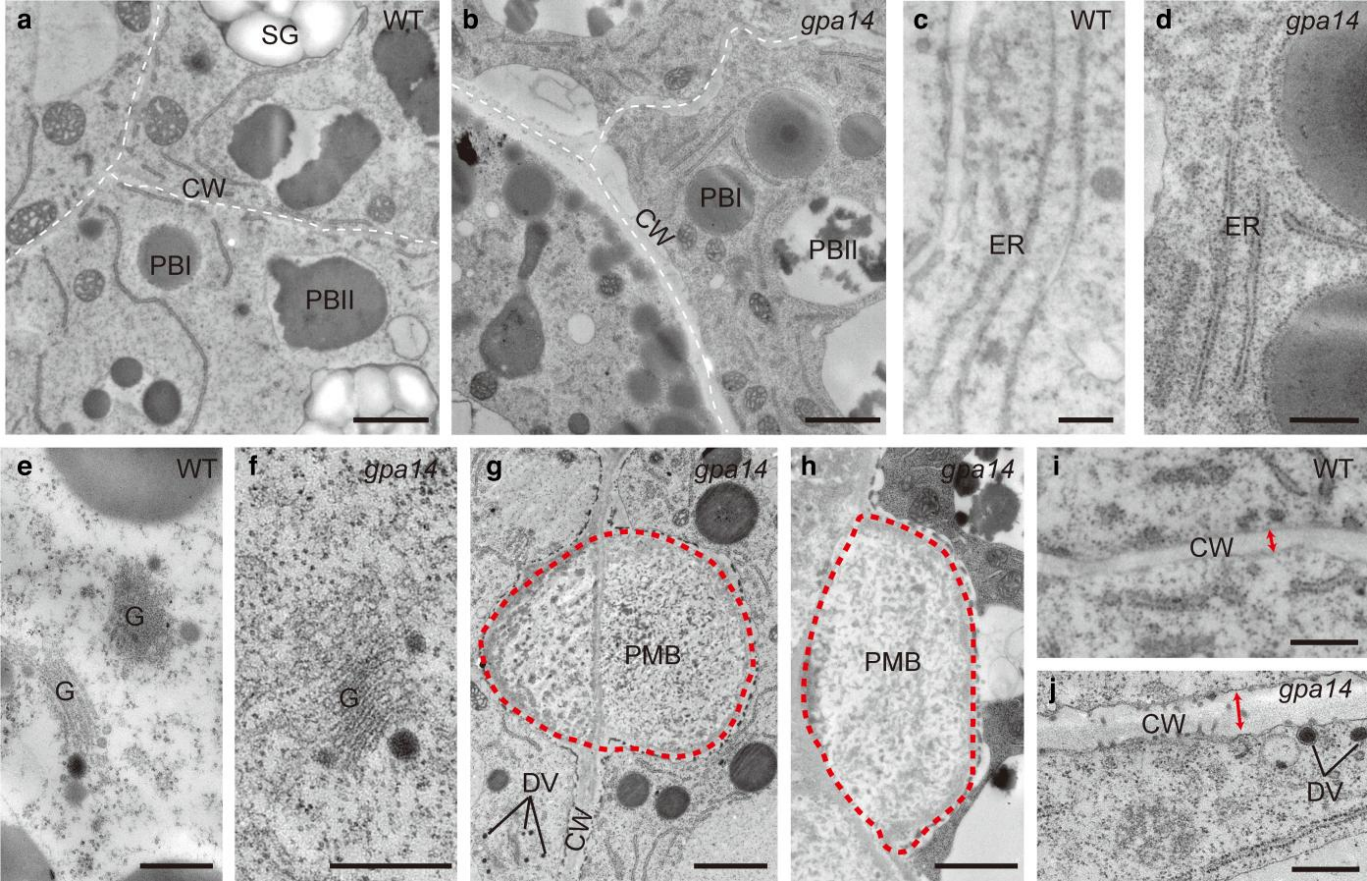
TEM analysis of sub-aleurone layer cells in the developing endosperm of WT and *gpa14* mutant. **a–b)** TEM images showing PBⅠs and PBⅡs in sub-aleurone layer cells of 12 DAF endosperm in WT (a) and *gpa14* mutant (b). Abbreviations: CW, cell wall; PBⅠ, protein body Ⅰ; PBⅡ, protein body Ⅱ; SG, starch granule. **c–d)** ER morphology in WT (c) and *gpa14* mutant (d). ER, endoplasmic reticulum. **e–f)** Golgi morphology in WT (e) and *gpa14* mutant (f). G, Golgi apparatus. **g–h)** Presence of PMB structures in *gpa14*. The red dotted line marks the PMB. **i–j)** Cell wall structures in WT and *gpa14* mutant. The red bidirectional arrow indicates the thickness of the cell wall. Abbreviations: CW, cell wall; DV, dense vesicle. Scale bars: 2 µm for panels a, b and g; 500 nm for panels c–f and h–j.

To better understand the ultrastructural effects of the *gpa14* mutation, we performed transmission electron microscopy (TEM) to compare subcellular structures in developing endosperm at 12 DAF. In WT, we observed well-filled, irregularly shaped PBIIs (3 to 4 µm in diameter) and spherical PBⅠs (1 to 2 µm in diameter) surrounded by rough endoplasmic reticulum (RER) (Fig. 3a). Similarly, in *gpa14* endosperm, spherical PBⅠs surrounded by RER were present. However, we observed thickened cell walls and incompletely filled PBⅡs compartments (Fig. 3b). The morphology of the ER showed no noticeable differences between WT and *gpa14* (Fig. 3c, d). DVs are known to be key carriers for sorting of storage proteins which are approximately 100 to 200 nm in diameter (Wang et al. 2010; Liu et al. 2013; Zhang et al. 2025). DVs were budding from the TGN were detected in both WT and *gpa14* mainly according to the vesicle's diameter and subcellular location (Fig. 3e, f). However, compared with WT, some DVs in *gpa14* were missecreted around the plasma membrane (PM), and electron-dense swollen structures were observed in the extracellular spaces (ECS) adjacent to the cell walls (Fig. 3g–j). These electron-dense structures, known as PMBs, resemble those previously identified in 57H mutants *gpa1*, *gpa3*, *gpa5*, and *gl*up6 (Wang et al. 2010; Liu et al. 2013; Wen et al. 2015; Ren et al. 2020). Combining these observations with results from double immunofluorescence labeling (Fig. 2c–j), we suggested that PMB structures in the *gpa14* mutant result from the missorting of glutelins, globulin and cell wall components into the ECS (Fig. 3i, j). Furthermore, given that BiP1 and PDI1-1 protein levels in *gpa14* mutant were comparable to those in WT (Fig. 1l), we propose that the accumulation of 57-kDa glutelin precursors in the *gpa14* mutant arises from the defects in DV-mediated post-Golgi trafficking.

### Map-based cloning of the gene responsible for the *gpa14* phenotype

**Figure 4:**
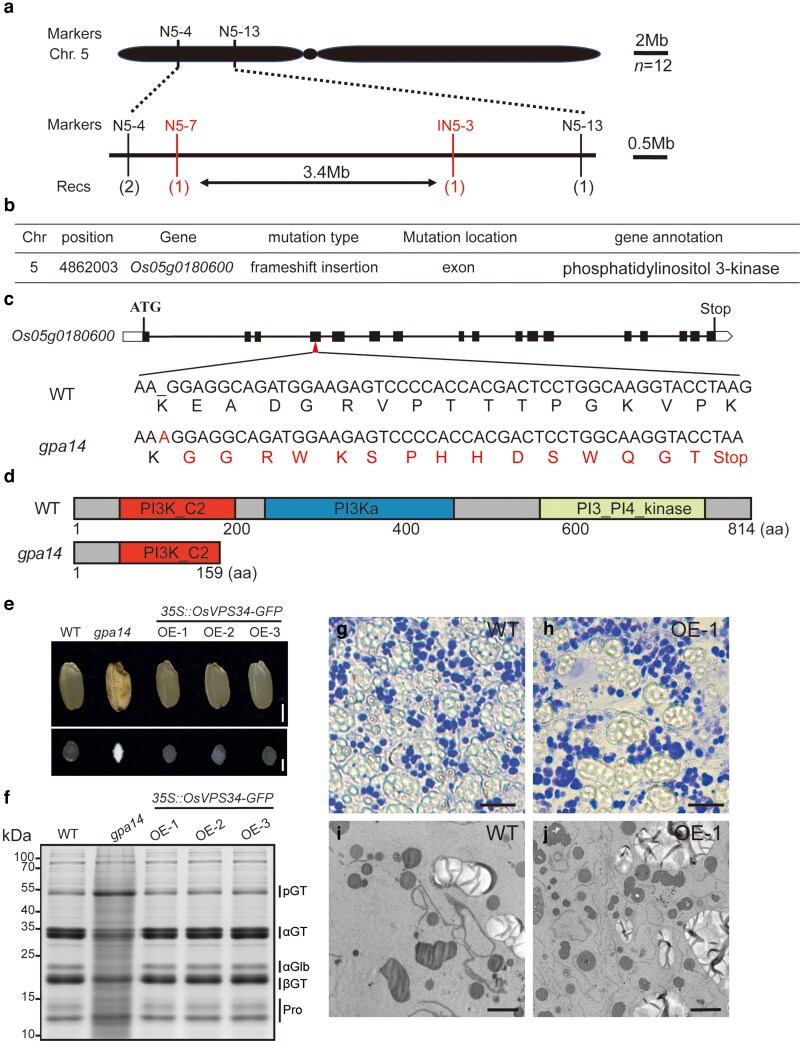
Map-based cloning of *GPA14*. **a)** The *GPA14* locus was initially mapped to 2.1 to 5.5 Mb region on chromosome 5 between the markers N5-7 and IN5-3. **b)** Whole-genome variation analysis through resequencing identified a single nucleotide insertion in an exon within the primary mapping interval. **c)** Schematic representation of the *Os05g0180600* gene structure and its mutation in the *gpa14* mutant. White boxes denote untranslated regions (UTRs), black boxes indicate exons, and lines represent introns. The ATG and stop codons mark translation start and stop sites, respectively. **d)** Protein structure comparison of OsVPS34 between WT and *gpa14*. In *gpa14* mutant, partial PI3K_C2, the entire PI3Ka and PI3_PI4_kinase domain are deleted. **e)** Phenotypes of mature grains from WT, *gpa14* mutant, and here *35S::OsVPS34-GFP* transgenic lines (OE-1, OE-2, OE-3). The top row shows intact brown rice grains, and the bottom row shows transverse cross-sections of the corresponding grains. Scale bars = 2 mm. **f)** Seed storage protein profile of mature seeds from WT, *gpa14* mutant, and 3 transgenic lines visualized by CBB staining of an SDS-PAGE gel. pGT, unprocessed glutelin precursors; αGT, mature acidic subunits of glutelin; αGlb, α-globulin; βGT, mature basic subunits of glutelin; Pro, prolamins. **g–h)** CBB staining of semithin sections of developing endosperm in WT and transgenic complementary line OE-1. Note that the image of WT shown in (g) is taken from the area enclosed by the green dashed box in Fig. 2a. Scale bars = 10 µm. **i–j)** TEM images of developing endosperm in WT and the transgenic line OE-1. Scale bars = 2 µm.

To investigate the genetic basis control underlying the *gpa14* phenotypes, we conducted segregation ratio analysis and map-based cloning. An F_2_ population was generated by crossing the heterozygous *gpa14* (+/−) mutant and the *indica* variety N22. Through SDS-PAGE analysis, we identified 12 individuals from the F_2_ population that exhibited 57H phenotype with floury, shrunken endosperm. DNA was extracted from these seeds for primary gene-mapping, and the *GPA14* locus initially was localized to a 2.1- to 5.5-Mb region on chromosome 5 between the markers N5-7 and IN5-3 (Fig. 4a). Due to the limited number of recessive individuals in the F_2_ population, we performed de novo sequencing analysis to identify variations between WT and *gpa14*. A single thymine insertion was identified in the fourth exon of *Os05g0180600* in the initial mapping interval, which resulted in a premature stop codon of this gene (Table S1; Fig. 4b–c). *Os05g0180600* encodes phosphatidylinositol 3-kinase, known as VPS34 based on its yeast ortholog. OsVPS34 consists of 814 amino acids with the PI3K_C2, PI3Ka, and PI3_PI4_Kinase domains. The *gpa14* mutation led to the deletion of the C terminal 655 amino acids (Fig. 4d).

To confirm that the mutation in *Os05g0180600* is responsible for the phenotypes in *gpa14*, we generated transgenic lines expressing *35S::OsVPS34-GFP* using seeds from *gpa14* heterozygous plants. In the T_2_ plants, we isolated individuals carrying both the *gpa14* (−/−) genotype and the *35S::OsVPS34-GFP* cassette (Fig. S2). The transgenic plants exhibited a restored transparent endosperm phenotype (Fig. 4e). SDS-PAGE analysis demonstrated that glutelin precursor levels were reduced to WT levels in the positive transgenic plants (Fig. 4f). Additionally, cytological observations revealed that the disappearance of PMB structures and the restoration of normal PBⅡ filling, resembling those of WT (Fig. 4g–j). Therefore, we suggested that *Os05g0180600*, designated *OsVPS34*, is responsible for the glutelin precursor accumulation phenotype in the *gpa14* mutant.

### Expression pattern and subcellular localization of OsVPS34

**Figure 5:**
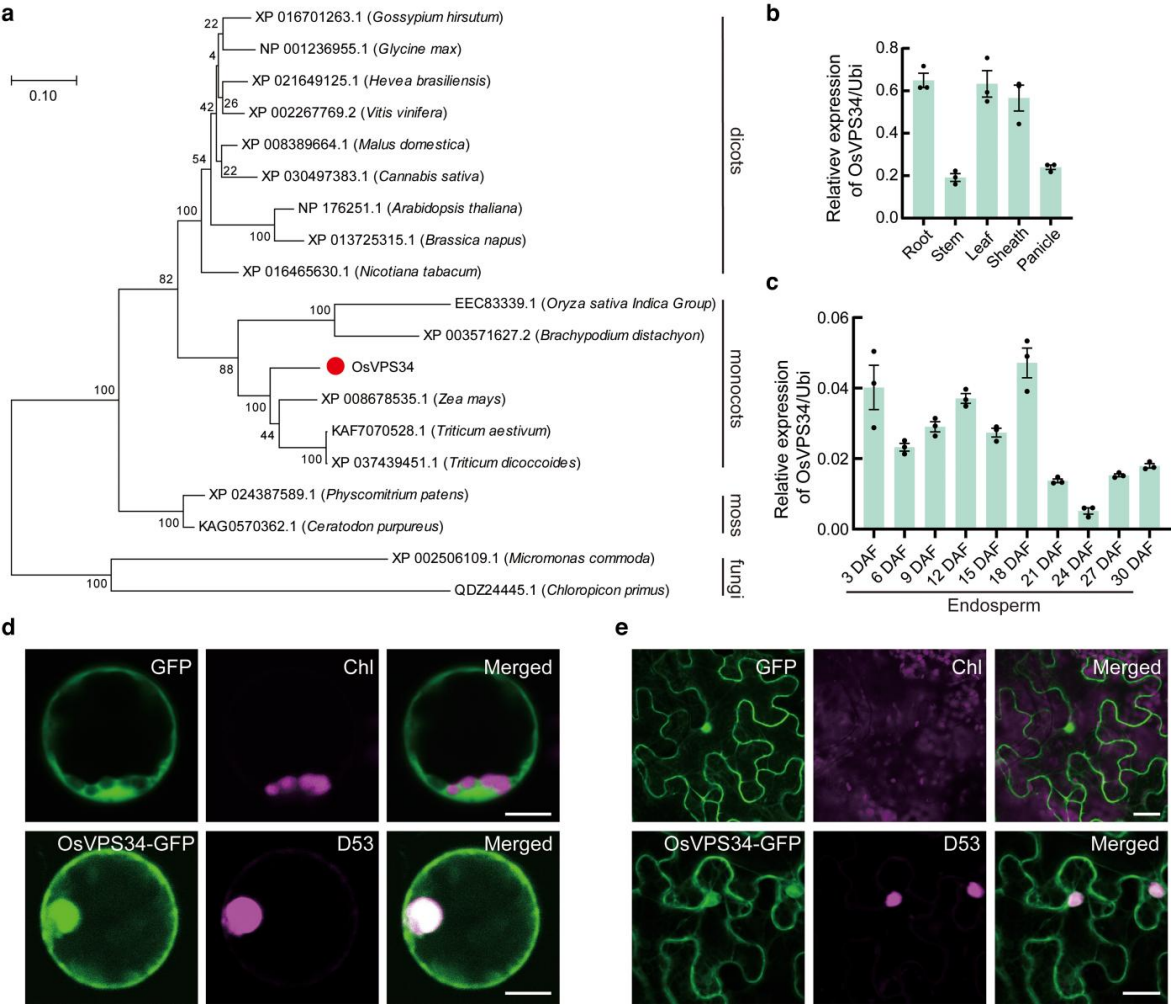
Phylogenetic, expression, and subcellular localization analyses of *OsVPS34*. **a)** Phylogenetic tree of OsVPS34 and its homologs. The tree was constructed using MEGA 7.0 with 1,000 bootstrap replicates. Proteins are named according to NCBI accession numbers, with species names indicated in parentheses. The red dot highlights OsVPS34. Numbers at the nodes indicate bootstrap values. The scale bar represents 0.10 substitutions per site. **b–c)** Expression profiles of *OsVPS34* in root, stem, leaf, sheath, panicle (b) and developing endosperm (sampled every 3 d from 3 to 30 DAF) (c). Data are presented as mean ± SD from 3 biological replicates (*n* = 3), with *Ubiquitin* (*Ubi*) serving as internal control. **d)** Subcellular localization of OsVPS34-GFP in rice protoplasts observed by laser confocal microscopy. Chlorophyll autofluorescence (Chl) and nuclear marker D53-mCherry are magenta. Scale bars = 10 µm. **e)** Laser confocal microscopy images showing the subcellular localization of OsVPS34-GFP in leaf epidermal cells of *Nicotiana benthamiana*. Empty GFP was used as the control, Chlorophyll autofluorescence (Chl) and nuclear marker D53-mCherry are magenta. Scale bars = 25 µm.

To further investigate the molecular function of *OsVPS34*, we first conducted a phylogenetic analysis. The phylogenetic analysis revealed that OsVPS34 is conserved among dicots, mosses, and monocots, with the closest homologs identified in wheat and maize (Fig. 5a). Protein sequence alignment demonstrated that PI3K_C2, PI3Ka, and PI3_PI4_Kinase domains are highly conserved across different species (Fig. S3). Then, we carried out RT-qPCR to examine the expression pattern of *OsVPS34* during the heading stage and filling stage. The results indicated that *OsVPS34* is constitutively expressed during the heading stage, with the lowest expression observed in stem (Fig. 5b). Although *OsVPS34* exhibited lower expression levels in the endosperm but relatively higher in the developing endosperm from 3 to 18 DAF, a critical period for endosperm development, especially for starch and protein synthesis (Fig. 5c). These findings suggest that OsVPS34 may play an important role in endosperm development.

In yeast, Vps34p localizes to the pre-autophagosomal structure (PAS), vacuolar membrane, and endosomes (Obara et al. 2006). In mammals, VPS34 is predominantly detected in early endosomes, lysosomes, autophagosome, the ER, and the Golgi apparatus (Backer 2008). In *Arabidopsis*, VPS34 is mainly localized in early endosomes (Itakura et al. 2008; Wang et al. 2023). To determine the subcellular localization of OsVPS34 in rice, we generated an OsVPS34-GFP fusion protein driven by the cauliflower mosaic virus 35S (CaMV 35S) promoter, containing the full-length coding region. In rice protoplasts, OsVPS34-GFP was detected in both the cytoplasm and nucleus, as evidenced by its co-localization with D53-mCherry, a nuclear localization marker (Fig. 5d). Similarly, when expressed in the leaf epidermal cells of *Nicotiana benthamiana*, OsVPS34 was also localized to both the cytoplasm and nucleus (Fig. 5e). As reported, VPS34 protein shows puncta signal in cytoplasm; OsVPS34 did not exhibit puncta localization pattern but rather a diffuse fluorescent signal. This may be caused by overexpression of OsVPS34 in protoplast and the leaf epidermal cells of *Nicotiana benthamiana* driven by 35S promoter.

### OsVPS34 is a component of the PI3K complex

**Figure 6:**
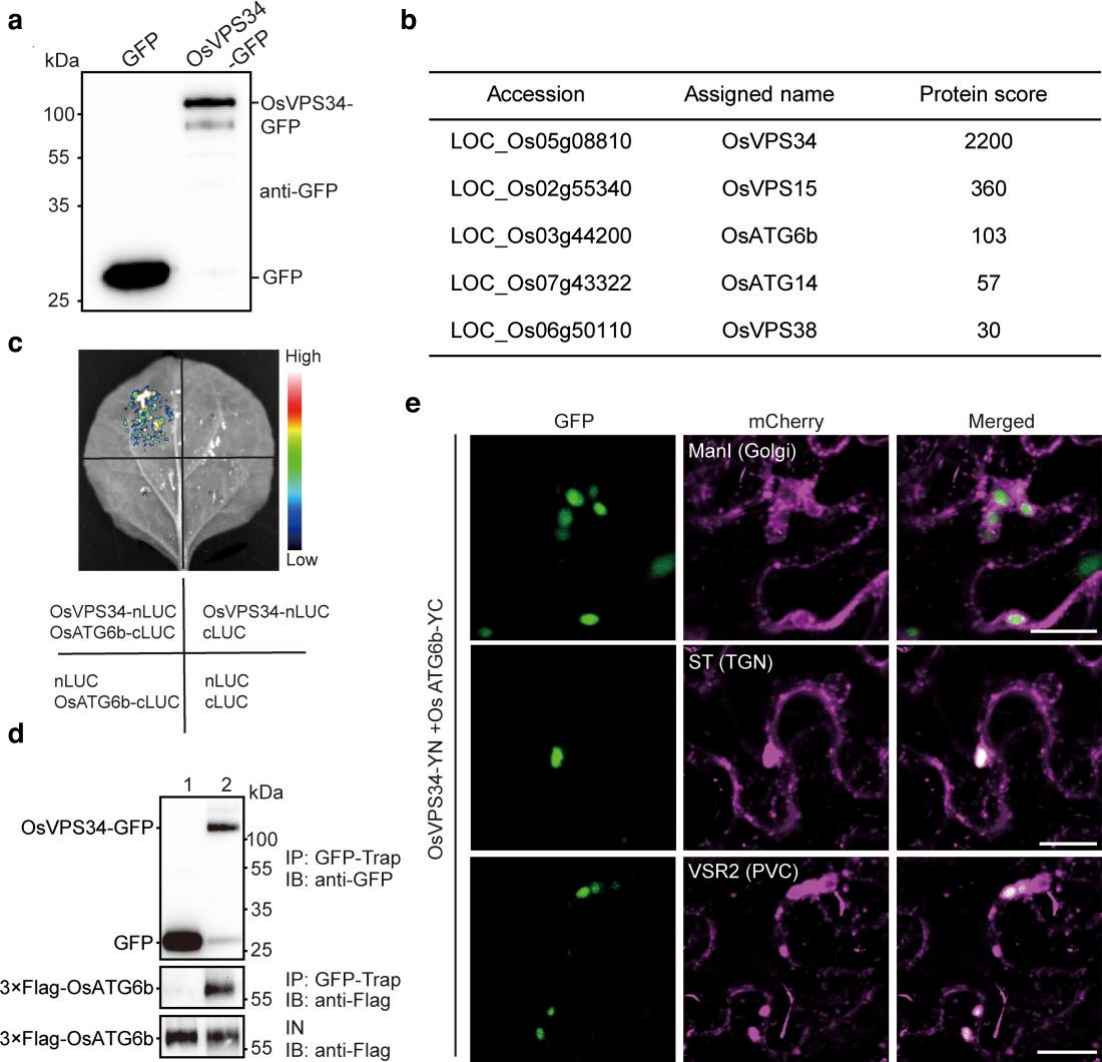
Verification of PI3K complex interactions in rice. **a)** Immunoblot analysis of precipitated proteins using GFP beads and detected with anti-GFP antibodies. **b)** Identification of OsVPS34-interacting proteins through mass spectrometry analysis. Protein scores were calculated using Mascot. No PI3K complex members were precipitated with free GFP. **c)** Firefly LCI assay showing the interaction between OsVPS34 and OsATG6b in *Nicotiana benthamiana* leaves. The colored scale bar represents luminescence intensity in counts per second. cLUC, C-terminal fragment of LUC; nLUC, N-terminal fragment of LUC. **d)** Co-IP assay confirming the interaction between OsVPS34 and OsATG6b in *Nicotiana benthamiana* leaves. 3×Flag-OsATG6b was transiently co-expressed with OsVPS34-GFP (lane 2) or free GFP (lane 1). 3×Flag-OsATG6b was co-immunoprecipitated by OsVPS34-GFP but not by free GFP using anti-GFP magnetic beads. IN, input; IB, immunoblot; IP, immunoprecipitation. **e)** Confocal microscopy images showing partial localization of the PI3K complex to the TGN and PVC. OsVPS34-YN and ATG6b-YC were co-expressed with Man1-mCherry, ST-mCherry, and mCherry-AtVSR2 in *Nicotiana benthamiana* leaf cells. Scale bars = 25 µm.

In plants, PI3K complexes are classified into 2 types based on their functions and subunit composition: PI3K complex Ⅰ and PI3K complex Ⅱ (Liu et al. 2020). Although the PI3K complex has been extensively studied, how it assembles in crops such as rice, wheat, and maize remains unclear (Kihara et al. 2001; Balla 2013; Ohashi et al. 2019; Wang et al. 2022). To identify the components of the PI3K complex in rice, we performed an immunoprecipitation-mass spectrometry (IP-MS) analysis using 15-d-old seedlings overexpressed expressing OsVPS34-GFP, empty GFP as a control. After GFP-bead enrichment, an approximately 120-kDa band corresponding to OsVPS34-GFP was detected in the *35S::OsVPS34-GFP* transgenic line, whereas only a 27-kDa GFP band was observed in the *35S::GFP* control line (Fig. 6a, Fig. S4). Multiple potential interacting proteins of OsVPS34 were identified via IP-MS analysis, including OsVPS15, OsATG6b, OsVPS38, and OsATG14, which are the orthologs of PI3K components (Fig. 6b and Tables S2 and 3).

To further validate their interactions, we conducted yeast 2-hybrid (Y2H) assays. In the rice genome, we identified 1 ortholog for each OsVPS15, OsVPS38, and OsATG14, as well as 3 orthologs of OsATG6, which we designated as OsATG6a, OsATG6b, and OsATG6c. Due to the longer coding sequence of *OsVPS15* coding sequence, we failed to amplify it. The interaction between OsVPS34 and OsATG6b was the strongest (Fig. S5). All firefly luciferase complementation imaging (LCI), bimolecular fluorescence complementation (BiFC), and co-immunoprecipitation (Co-IP) assays in *Nicotiana benthamiana* confirmed the interaction between OsVPS34 and OsATG6b (Fig. 6c–e). Notably, coexpression of OsVPS34 and OsATG6b resulted in dot-like fluorescence signals (Fig. 6e; Fig. S6). Given that loss of OsVPS34 function disrupted storage protein trafficking at the post-Golgi stage in *gpa14* and that VPS34 has been reported to localize to early endosomes, lysosomes, and autophagosome (Obara et al. 2006; Backer 2008; Itakura et al. 2008; Wang et al. 2023), we further investigated the subcellular localization of the OsVPS34-OsATG6b complex. Using BiFC assays, we coexpressed OsVPS34 and OsATG6b with AtMan1-mCherry, AtST-mCherry, and AtVSR2-mCherry, which are markers for the Golgi, TGN, and PVC, respectively, in *Nicotiana benthamiana* leaf epidermal cells. The results showed that the OsVPS34-OsATG6b complex co-localizes with the TGN and PVC (Fig. 6e). These findings indicate that OsVPS34 is a key component of PI3K complex, and the OsVPS34-OsATG6b complex localizes to the TGN and PVC.

### Biological functions of the PI3K complex and subcellular localization of PI3P

**Figure 7:**
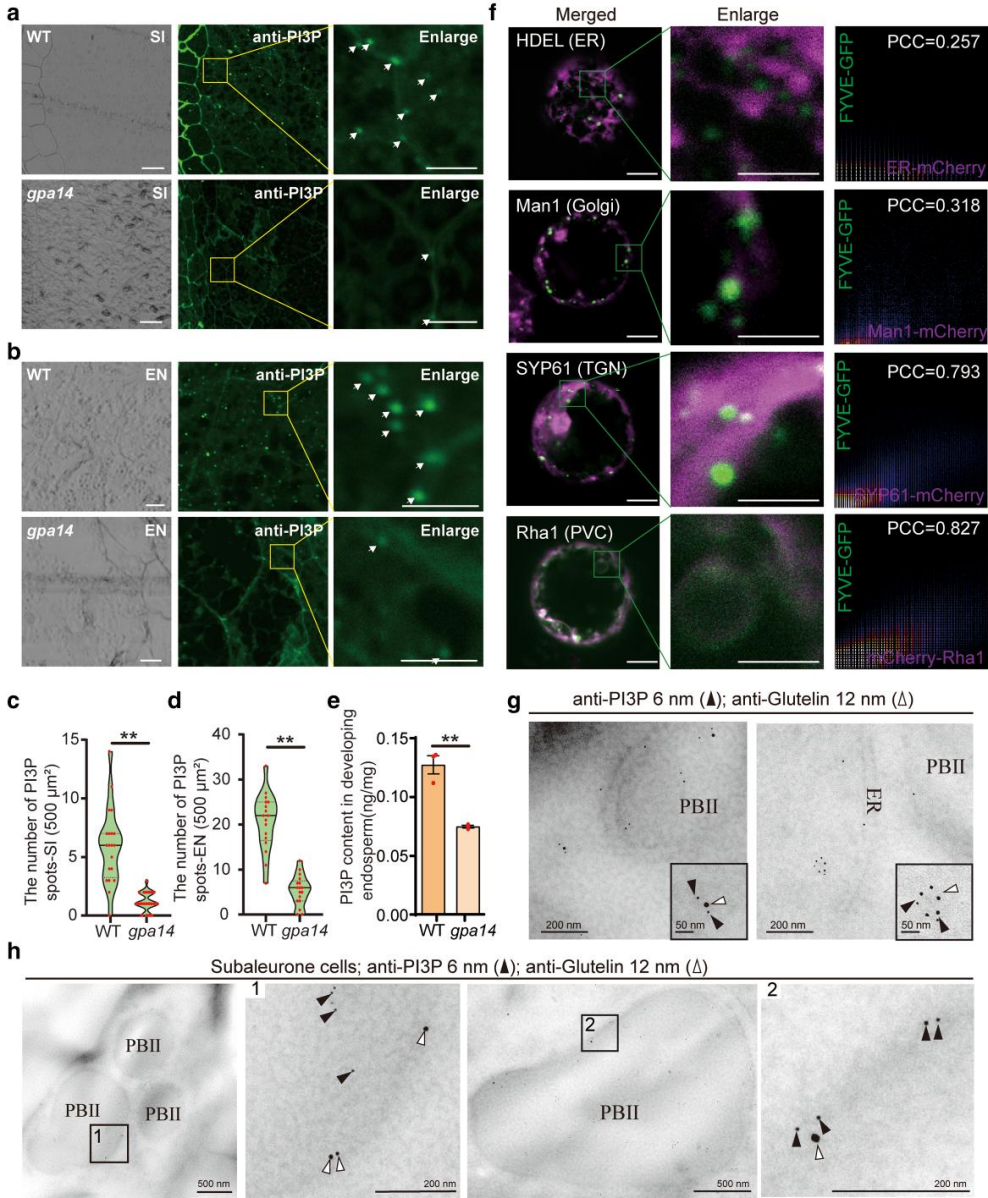
PI3P content and localization in rice endosperm. **a)** Immunofluorescence microscopy of PI3P in sub-aleurone cells of WT and *gpa14* mutant. Scale bars = 25 µm; enlarge bars = 10 µm. **b)** Immunofluorescence microscopy of PI3P in endosperm cells of WT and *gpa14* mutant. Scale bars = 10 µm; enlarge bars = 5 µm. Abbreviations: EN, endosperm cells; Sl, sub-aleurone cells. Anti-PI3P, PI3P-specific antibody. The white arrows indicate the point location of PI3P in endosperm cells. **c, d)** Quantification of PI3P fluorescent puncta in sub-aleurone cells (c) and endosperm cells (d) of WT and *gpa14* mutant. In the violin plots, the outlines represent the probability density of the data distribution, and the red dots represent individual biological data points. The solid and dashed horizontal lines within the plots indicate the medians and quartiles (Q1 and Q3), respectively. Asterisks indicate statistical significance determined by Student's *t*-tests (*n* ≥ 15; *P < 0.05, ** *P* < 0.01). **e)** PI3P content in developing endosperm of WT and *gpa14* mutant. Error bars represent the standard deviation (SD). Red dots indicate individual biological replicates. Asterisks indicate statistical significance determined by Student *t* tests (*n* = 3; **P < 0.01). **f)** Subcellular localization of PI3P using FYVE-GFP in rice protoplasts. FYVE-GFP (green) was transiently co-expressed with organelle markers (magenta): HDEL-mCherry (ER marker), Man1-mCherry (Golgi marker), SYP61-mCherry (TGN marker), and mCherry-Rha1 (PVC marker). Co-localization was analyzed using confocal microscopy. The scatterplots on the right represent the intensity distribution of all pixels in the 2 fluorescence channels. A greater degree of co-localization is indicated by a more diagonal scatterplot pattern. Scale bars = 10 µm in merged images and 5 µm in enlarged images. **g–h)** Immunogold labeling of ultra-thin sections of WT endosperm at 12 DAF using PI3P- and glutelin-specific antibodies. Glutelin antibodies are labeled with 12-nm gold particles (indicated by white arrowheads), while PI3P antibodies are labeled with 6-nm gold particles (black arrowheads). PI3P was detected in PBIIs (g) and DVs (f). Magnified views of regions 1 and 2 are shown. Scale bars are marked in each figure.

The PI3K complex phosphorylates PI to produce PI3P, as previously reported (Backer 2008; Simonsen and Tooze 2009). To investigate the biological function of the OsVPS34-mediated PI3K complex in rice, we performed immunofluorescence labeling using commercial PI3P-specific antibody on semi-thin sections of the WT and *gpa14* mutant endosperm at 12 DAF. The anti-PI3P antibody has been reported previously shown to exhibit a punctate localization pattern in immunofluorescence labeling (Devanathan et al. 2020; Ray et al. 2022; Wrobel et al. 2022; Kang et al. 2024; Elisabeth et al. 2025). Consistently, we observed PI3P puncta signals in both sub-aleurone layer (Sl) cells and endosperm (EN) in WT (Fig. 7a–b). However, the number of punctate structures were markedly reduced in the *gpa14* mutant (Fig. 7a, b). Statistical analysis confirmed the reduction of PI3P puncta signals in the *gpa14* mutant seeds (Fig. 7c, d). Meanwhile, we also measured the PI3P content in developing endosperm of WT and *gpa14* mutant, which showed that the PI3P content in the *gpa14* was significantly decreased (Fig. 7e). These results suggest that OsVPS34 plays a crucial role in the PI3K complex-mediated production of PI3P in rice.

PI3P is an essential membrane signaling molecule involved in endocytosis, endosomal sorting, and macro-autophagy (Noack and Jaillais 2017; Di Sansebastiano et al. 2018; Marshall and Vierstra 2018). In plants, PI3P has been shown to accumulate in late endosomes/PVC using a 2×FYVE reporter (Vermeer et al. 2006; Simon et al. 2014). To further investigate the subcellular localization of PI3P in rice, we constructed a FYVE-GFP fusion protein to track its distribution (Fig. S7a) (Simon et al. 2014). In rice protoplasts, FYVE-GFP displayed a dot-like pattern in the cytoplasm, while dot-like signal of FYVE-GFP dispersed and the PI3P content markedly decreased while treated with LY294002 (a PI3K inhibitor) (Fig. S7e). And the abundance of FYVE-GFP was decreased by LY294002 treatment (Fig. S7c–d). Based on these results, we coexpressed FYVE-GFP with various organelle marker proteins, including HDEL-mCherry (ER marker), Man1-mCherry (Golgi marker), mCherry-SYP61 (TGN marker), and mCherry-Rha1 (PVC marker). Co-localization analysis revealed that PI3P predominantly localizes to the TGN and PVC as OsVPS34-OsATG6b complex, with a Pearson correlation coefficient (PCC) of 0.793 and 0.827, respectively (Figs. 5e, 7f and Fig. S7f). To gain further insights into PI3P localization in developing rice endosperm and its role in glutelin sorting, we conducted immunogold labeling on ultra-thin sections of WT endosperm at12 DAF using PI3P- and glutelin-specific antibodies. PI3P was labeled with 6-nm gold particles, while glutelin was labeled with 12-nm gold particles. The results showed that PI3P co-localized with glutelin in DVs and PBIIs (Fig. 7f–g). These findings suggest that PI3P functions as a key membrane signaling molecule, coordinating SSPs transport from DVs to PSVs/PBIIs in rice endosperm.

### Downregulation of intracellular PI3P levels results in abnormal subcellular localization of Rab5a and GPA5

**Figure 8:**
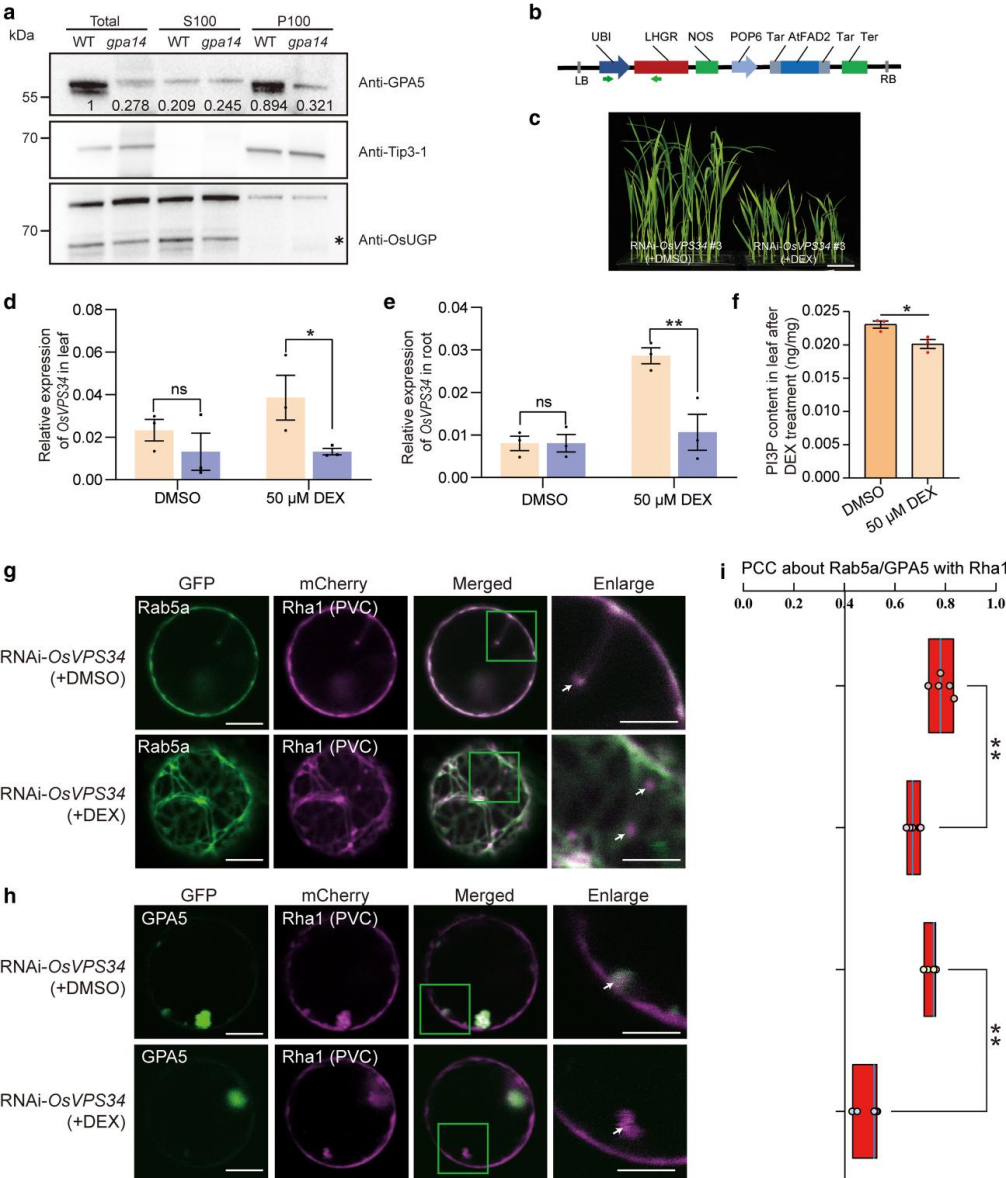
Localization analysis of Rab5a and GPA5 upon disruption of PI3K complex function. **a)** Immunoblot analysis of GPA5 in the membrane (P100) and cytoplasmic (S100) fractions. Total protein was extracted from 12 DAF developing endosperm of WT and *gpa14* and subjected to ultracentrifugation (100,000 × *g* for 1 h) to separate P100 (membrane fraction) and S100 (cytoplasmic fraction). Anti-Tip3-1 was used as a P100 marker, and anti-OsUGP as a S100 marker. Asterisks indicate OsUGP bands. **b)** The diagram of DEX RNAi-*OsVPS34* vector. The green arrow indicates the position of the PCR identification primers. **c)** Observation after DEX treatment of the RNAi-*OsVPS34* seedlings. DMSO was used as control. Scale bar = 5 cm. **d, e)** Detection of OsVPS34 expression levels in the leaves (d) and roots (e) of WT and RNAi-*OsVPS34* seedlings before and after DEX treatment. Data represent mean ± SD (*n* = 3; ns *P* > 0.05; *P < 0.05, **P < 0.01), evaluated by Student *t* test. **f)** Determination of PI3P content in the leaves of seedlings after DEX treatment. Data represent mean ± SD (*n* = 3; *P  *<* 0.05, Student *t* test). **g, h)** Co-localization of Rab5a-GFP (green) and GPA5-GFP (green) with the PVC marker mCherry-Rha1 (magenta) in rice protoplasts extracted from RNAi-*GPA14* seedlings. DMSO treatment was used as a control. Scale bars = 10 µm, enlarge bars = 5 µm. **i)** Higher PCC values indicate stronger co-localization, while lower values indicate reduced co-localization. In the box plots, the center line represents the median, the box limits represent the upper and lower quartiles, the whiskers represent 1.5× the interquartile range, and the points represent outliers and individual measurements. Statistical significance was determined by Student *t* test (*n* = 5; **P < 0.01).

GPA5, a key regulator of glutelin transport from DVs to PBIIs, acts as an effector of Rab5a and specifically binds to PI3P via its PX domain (Wang et al. 2010; Ren et al. 2020). To investigate how PI3P levels affect the subcellular location of GPA5 and Rab5a, we first coexpressed Rab5a-GFP and GPA5-GFP with FYVE-mCherry, respectively. Both Rab5a and GPA5 co-localized with FYVE-mCherry, which is consistent with a previous report (Fig. S8) that PI3P is an important anchor signal for PVC and TGN subcellular location of Rab5a-GFP and GPA5-GFP (Ren et al. 2020). To further verify the effect of the decrease in PI3P level on the localization of GPA5 and Rab5a, we conducted a component separation experiment with *gpa14* homozygous seeds, which isolated and genotyped from the heterozygous *gpa14* (+/−) individual. Anti-Tip3-1 and anti-OsUGP were used as marker proteins for the P100 (membrane fraction) and S100 (cytoplasmic fraction), respectively. Using immunoblotting with a GPA5 antibody, we found that total and membrane-associated GPA5 protein levels were reduced in the *gpa14* mutant (Fig. 8a). Because the customized antibody of Rab5a did not work, we carried out the component separation experiment for Rab5a with 15-d-old Rab5a-GFP transgenic seedlings treated with 50 µM LY294002. Using immunoblotting with GFP and GPA5 antibody, we can see the proportions of both Rab5a and GPA5 in the membrane fraction substantially decreased, whereas their presence in the cytoplasmic fraction increased (Fig. S9a–c).

**Figure 9:**
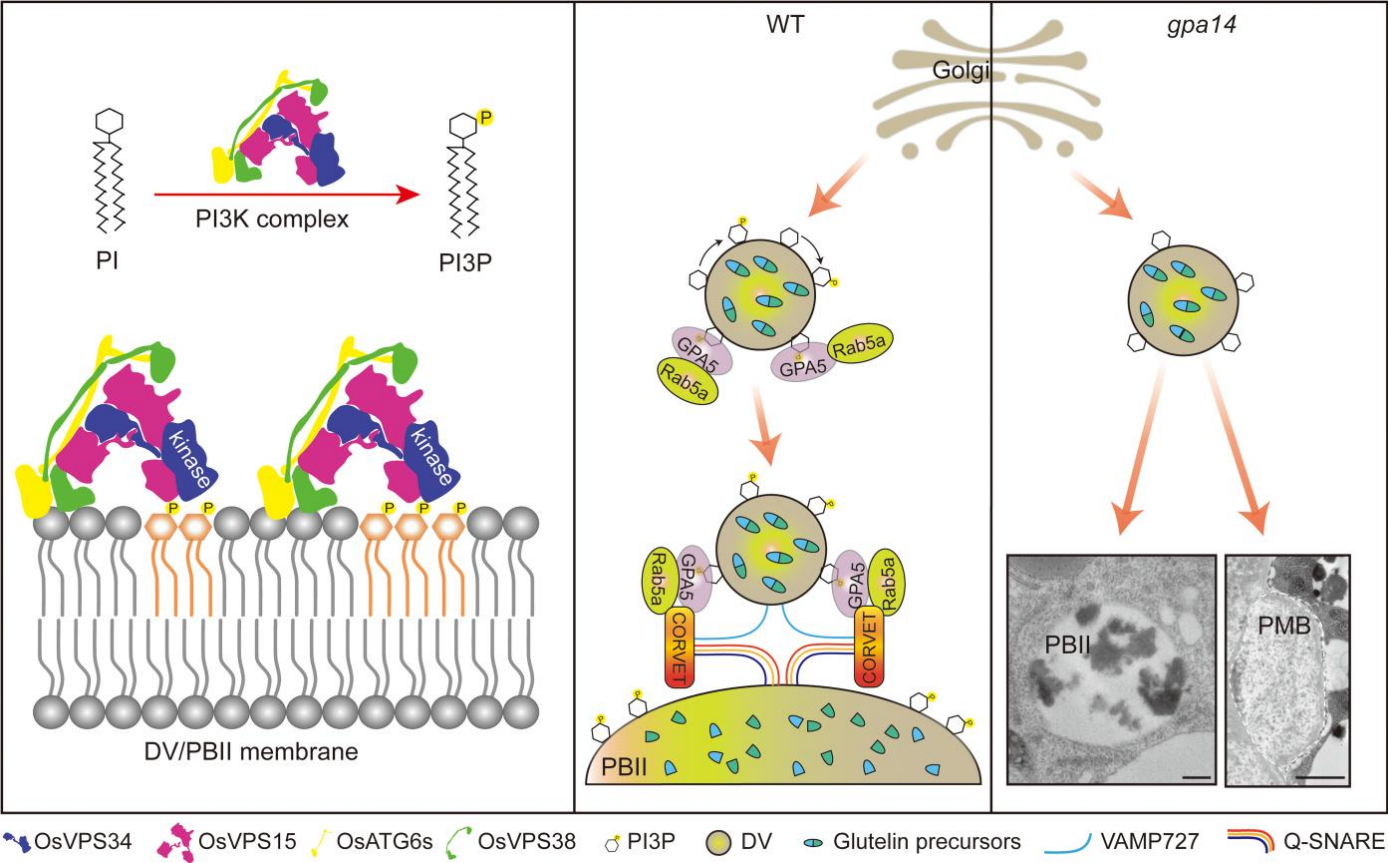
A working model illustrating the role of OsVPS34 in post-Golgi trafficking of glutelin in developing rice endosperm. In the WT, OsVPS34 functions as a subunit of the PI3K complex, catalyzing the phosphorylation of PI on DV/PBⅡ to generate PI3P (left panel). PI3P subsequently recruits GPA5, which interacts with Rab5a. Subsequently, GPA5 interacts with the class C core vacuole/endosome tethering (CORVET) complex and the VAMP727-coatining soluble N-ethylmaleimide sensitive factor attachment protein receptor (SNARE) complex to mediate DV-PBⅡ fusion (Ren et al. 2020). However, in the *gpa14* mutant, defective kinase activity in the PI3K complex prevents the production of PI3P on DV/PBⅡ, leading to improper localization of GPA5 and Rab5a. As a result, DVs cannot be correctly transported to PBIIs after budding from the TGN, resulting in inadequately filled PBIIs or PMB structures. The structure of PI3K complex was referred to previous studies (Ohashi 2021). These images are the same as those shown in Fig. 3b, h. Scale bars = 500 nm.

To overcome the lethality, we constructed *DEX-RNAi-OsVPS34* transgenic plants, which could downregulate the expression of *OsVPS34* by dexamethasone (DEX)-inducible RNAi (Fig. 8b). The positive transgenic plants were identified through PCR (Fig. S10). After treating *DEX-RNAi-OsVPS34* transgenic plants with DEX (50 µM DEX, 5 to 7 d), we found the seedling was shorter and expression of *OsVPS34* and content of PI3P significantly decreased compared with treatment with DMSO (Fig. 8c–f). Based on these data, we co-repressed Rab5a and GPA5 with PVC markers Rha1 in the protoplast of DEX-RNAi-*GPA14* transgenic seedlings treated with DEX and DMSO separately. We found that the co-overlapping signals between Rab5a-GFP and GPA5-GFP with mCherry-Rha1 significantly decreased in protoplast treated with DEX compared with it treated with DMSO (Fig. 8g–i). These findings suggest that PI3P acts as a critical phospholipid signal, facilitating the recruitment of GPA5 and Rab5a onto PVCs/DVs (Fig. 9). This process is crucial for the proper trafficking of glutelin during the post-Golgi process.

## Discussion

### OsVPS34 plays a crucial role in post-Golgi trafficking of glutelin

In rice seeds, SSPs are synthesized on the ER and transported via the Golgi-dependent endomembrane trafficking system for deposition in protein bodies, kind of PSV (Ren et al. 2022). GPA5, localized to the TGN and PVC, could bind phosphatidylinositide-3-phosphate (PI3P) and is involve in Golgi-dependent endomembrane trafficking of SSP (Wang et al. 2010; Ren et al. 2020). Through map-based cloning, we demonstrated that the post-Golgi sorting defects observed in *gpa14* mutants result from impaired PI3P production due to loss of OsVPS34 function (Figs. 4, 6 and 7). Immunogold labeling revealed co-localization of PI3P and glutelin in DVs and PBII (Fig. 7g–h). Furthermore, cell fractionation and subcellular localization analyses of GPA5 and Rab5a confirmed that PI3P serves as the key phospholipid signal mediating post-Golgi sorting, directly influencing their organellar targeting and membrane association (Fig. 8a; Fig. S9). In *gpa14* homozygous endosperm, total GPA5 protein levels were reduced compared with wild type, primarily due to decreased membrane-associated pools (Fig. 8a). We propose that PI3P-dependent endomembrane localization maintains GPA5 protein abundance, though the molecular mechanism regulating its stability remains unknown. There are 2 types of vacuoles, the lytic vacuole (LV) and PSV, with different physiological functions. Both vacuoles show common trafficking mechanisms, but certain aspects are specific to trafficking to either the LV or PSV (Zhang et al. 2021). The function of PI3P, localized in vacuole membrane and late endosome/PVC and produced by PI3K, has been extensively studied in endosomal sorting, lysosomal/vacuolar transport, and autophagy. However, lethality of PI3K core components has limited investigations into PI3P roles in seed storage protein trafficking (Simon et al. 2014; Liu et al. 2018). Here, we successfully isolated homozygous *gpa14* developing seeds and mature grain from heterozygous plants (Figs. 2 and 3), enabling functional exploration of PI3P and PI3K during seed development. Our results highlight rice seeds as a unique and advantageous system for dissecting PI3K-mediated vesicle trafficking in developing seeds.

### Functional divergence of PI3K complex components in rice

The PI3K complex consists of the catalytic subunit VPS34 and 3 regulatory subunits that together form a canonical class III PI3K responsible for PI3P production (Safaroghli-Azar et al. 2023). VPS34 is evolutionarily conserved across eukaryotes and functions as the core kinase component (Safaroghli-Azar et al. 2023). Through IP-MS analysis of OsVPS34-GFP seedlings, we identified OsVPS15, OsATG6b, OsVPS38, and OsATG14 as interacting proteins (Fig. 6b; Tables S2 and S3). Among them, OsATG6b interaction was further validated by Y2H, LCI, BiFC, and Co-IP assays (Fig. S5; Fig. 6c–e). These results indicate that rice contains both OsATG14–OsVPS34 complex I and OsVPS38–OsVPS34 complex II. To dissect the functions of individual PI3K components, we generated CRISPR/Cas9 mutants of *OsVPS15*, *OsVPS38*, and *OsATG14*. However, only homozygous *Cr-osatg14* mutants were recovered, while neither homozygous nor heterozygous knockout lines were obtained for *OsVPS15* or *OsVPS38* (Figs. S11–S13). This is consistent with Arabidopsis, where null mutants of the 3 core PI3K components are sterile, while *atg14a atg14b* double mutants show relatively normal phenotypes (Liu et al. 2020). Arabidopsis *vps38* mutants are viable but display developmental defects in leaves, roots, and seeds (Liu et al. 2018). These phenotypic differences suggest potential functional divergence between PI3K complexes. In addition, the *Cr-osatg14* mutant thus provides valuable genetic material for investigating the function of the OsATG14–OsVPS34 complex I in autophagy. In addition, DEX-RNAi-*OsVPS34* transgenic plants exhibited reduced *OsVPS34* expression, shorter seedling growth, and decreased PI3P levels (Fig. 8b–f), offering an effective system for further dissecting PI3K roles during the vegetative stage.

### Subcellular localization and potential nuclear functions of OsVPS34

In yeast, mammals, and Arabidopsis, VPS34 predominantly localizes to early endosomes and is also detected in autophagosomes and Golgi-associated membranes (Obara et al. 2006; Backer 2008; Wang et al. 2023). In our study, OsVPS34-GFP was observed in both the cytoplasm and nucleus of rice seedling protoplasts and *N. benthamiana* leaf epidermal cells (Fig. 5d–e). Unlike previous reports, OsVPS34 did not form punctate structures when overexpressed alone, but instead exhibited diffuse fluorescence. However, coexpression of OsVPS34 and OsATG6b in BiFC assays produced punctate fluorescent signals that co-localized with TGN and PVC markers, consistent with PI3K complex functions in endosomal sorting, vacuolar transport, and post-Golgi SSP trafficking. We speculate that diffuse localization of OsVPS34 may result from overexpression under the 35S promoter, which could obscure its native patterns. In mammals, catalytically active PI3K also localizes to distinct nuclear subdomains, including regions associated with Pol I, Pol II, and Pol III transcription (Bunney et al. 2000). The nuclear PI3K pathway has been implicated in human diseases such as cardiovascular disorders and cancer (Davis et al. 2015). Vps34 can act as a transcriptional activator of p62 by competing with Nrf2 for Keap1 binding (Jiang et al. 2017), and Vps15 has been identified as a coactivator of the Bmal1-Clock transcription factor complex that regulates circadian metabolism (Alkhoury et al. 2023). These findings suggest that although the functions of VPS34 complexes in endosomal sorting and autophagy are well established, their nuclear roles remain largely unexplored. Although our observations indicate that OsVPS34 may be present in the nucleus, additional evidence, such as endogenous promoter-driven localization, nuclear fractionation, and functional assays, are required to verify its nuclear targeting and to clarify the potential nuclear functions of PI3K complexes in plants.

## Materials and methods

### Plant materials and growth conditions

The *gpa14* mutant was isolated from a mutagenesis library of the *Japonica cv.* Ningjing 4, generated through tissue culture. During the summer, plants were cultivated at the Tiao Qiao Breeding Base of Nanjing Agricultural University (32.0584°N, 118.7965°E) in Nanjing City, Jiangsu Province. In winter, they were grown at the Ling Shui Breeding Base of Nanjing Agricultural University (18.2525°N, 109.5121°E) in Sanya City, Hainan province. All samples for phenotypic analysis were collected in Nanjing.

### SDS-PAGE and immunoblot analysis

The methods for total seed protein extraction, SDS-PAGE and immunoblot analysis were performed as described in previous studies (Wang et al. 2010). The dilution ratio of polyclonal antibodies, including anti-glutelins, 10 kDa prolamin, α-globulin, BiP1, and PDI1-1, were consistent with those reported in prior studies (Wang et al. 2010; Ren et al. 2020). Anti-Actin (ABclonal, AC009) was used as a loading control. Anti-IgG (H + L chain) (Rabbit) pAb-HRP (MBL, 458) and Anti-IgG (H + L chain) (Mouse) pAb-HRP (MBL, 330) were used as secondary antibodies. SDS-PAGE and immunoblot analyses were repeated at least 3 times, and representative results were selected for presentation. All antibodies used in this study were diluted at a ratio of 1:5,000.

### Microscopy observation

The cross-sections of the mature seeds were observed using a HITACHI Regulus 8100 scanning electron microscope (Hitachi, Tokyo) after being coated with gold powder. For TEM, a 1-mm cross-section of developing seeds at 12 DAF was fixed in 1.25% glutaraldehyde and 1.25% paraformaldehyde in 0.2 M phosphate buffer (pH 7.2) for 24 h at 4 °C in dark. Ultra-thin sections were processed as described (Ren et al. 2020) using an ultramicrotome (Power Tome-XL; RMC, http://www.rmcproducts.com/). Images were captured using a Hitachi HT7800 transmission electron microscope (Hitachi, Tokyo). For semi-thin section preparation, 1-mm-thick sections from the middle part of developing seeds at 12 DAF were fixed, dehydrated through an ethanol gradient, and infiltrated with LR White resin (London Resin, Berkshire, UK) before embedding (Zhu et al. 2021). Semi-thin (1 μm) sections were stained with CBB following previously described methods (Ren et al. 2020) and observed under an optical microscope (Nikon ECLIPSE80i, Tokyo).

For immunofluorescence labeling experiments, the semi-thin (1 μm) sections were first incubated in TBST (pH 7.4, 0.5% Tween20) for 10 min, followed by blocking with 3% BSA in TBST. The sections were then incubated with the primary antibody diluted in blocking buffer (1% BSA in TBST), followed by 3 washes with TBST. Next, the samples were incubated with a combination of Alexa Fluor 488-conjugated (Invitrogen, A-21202) and Alexa Fluor 555-conjugated (Invitrogen, A-31572) secondary antibody and washed 3 times with TBST. Observations and imaging were performed using a LEICA TCS SP8 laser scanning confocal microscope. The primary antibody was diluted at a ratio of 1:100 (anti-Glutelin, anti-Prolamin, anti-Globulin and anti-PI3P), and the secondary antibody at 1:50. Alexa Fluor 488-conjugated secondary antibody (green fluorescence) was used to target glutelin, while Alexa Fluor 555-conjugated secondary antibody (magenta fluorescence) was used to target α-globulin and prolamins.

For immunogold labeling, ultra-thin sections were incubated in 3% BSA in PBS (pH 7.4) for 30 min. The sections were then incubated with the primary antibody (1:100) in 1% BSA in PBS, followed by 3 washes with 1% BSA in PBS. Next, the sections were incubated with 6-nm (Abcam, AB105276) or 12-nm (Abcam, AB105298) gold-conjugated secondary antibodies at 1:50 dilution and washed 3 times with 1% BSA in PBS. Finally, the sections were washed 5 times with ddH_2_O. Images were captured using a Hitachi HT7800 transmission electron microscope (Hitachi, Tokyo). Anti-PI3P (Z-P003) was purchased from ECHELON BIOSCIENCES.

### Map-based cloning of the *GPA14*

We crossed the *gpa14* (+/−) heterozygous plant (female parent) with the indica variety N22 (male parent) to generate hybrid combinations. The resulting F_1_ hybrids were grown at the breeding base in Lingshui, Hainan (18.2525°N, 109.5121°E), and F_2_ seeds were collected from individual F_1_ plants. The seeds exhibiting a floury and shrunken phenotype were isolated from F_2_ seeds and used for analysis of 57H over-accumulation. In detail, half of each seed, excluding the embryo, was ground into powder for total seed protein extraction, followed by SDS-PAGE analysis to observe the 57H precursor. The remaining half, which contained the embryo and exhibited an over-accumulation of 57-kDa glutelin precursor, was used for DNA extraction and genetic mapping. Additionally, we performed de novo sequence for both the WT and *gpa14* mutant to analyze variation and identify potential candidate genes within the initially mapped region. The molecular markers used for mapping are listed in Table S4.

### Genetic complementation analysis

The CDS sequence of *OsVPS34*, excluding the stop codon, was amplified from the seedling cDNA of Ningjing 4. This sequence was then inserted into the binary vector pCAMBIA1305, which had been linearized using SacI and BamHI, via homologous recombination (ABclonal, RM20523). The *35S::OsVPS34-GFP* construction was introduced into the callus produced by *gpa14* (+/−) mutant seeds through *Agrobacterium tumefaciens*-mediated transformation (Hiei et al. 1994). Positive transformants harboring *35S::OsVPS34-GFP* insertion were identified by PCR, and the genotype of *OsVPS34* were identified by sequencing (Table S4). The transformants with *35S::OsVPS34-GFP* insertion in *gpa14 (−/−)* were identified by phenotyping.

### Phylogenetic analysis

Homologous proteins of OsVPS34 across different species were identified using a BLASTP search on the NCBI website (http://www.ncbi.nlm.nih.gov/) and subsequently downloaded. Amino acid sequences were aligned using ClustalW, and a phylogenetic tree was constructed with MEGA7. The sequence alignment results were exported using ClustalX.

### Subcellular localization

The CDS sequence of *OsVPS34* excluding stop codon, was amplified from seedling cDNA of Ningjing 4 and inserted into pENTRY vector firstly via homologous recombination (ABclonal, RM20523). Subsequently, the CDS sequence of *OsVPS34* was recombined into the pAN580-gw1 vector (modified by our lab based on pAN580), driven the CaMV 35S promoter through an LR reaction (Invitrogen, 2269734). The full-length CDS of *D53* was cloned into the pAN583-mCherry vector driven by CaMV 35S promoter to generate a *D53*-mCherry fusion protein as a nuclear marker (Zhou et al. 2013). FYVE sequence was obtained from previous studies (Kim et al. 2001) and synthesized by GenScript. The FYVE sequence was amplified and inserted into pENTRY by homologous recombination and then recombined into the pAN580-gw1 vector via LR reaction to make *35S::FYVE-GFP* construction. The CDS of *AtARA7*, *AtRha1* were cloned from *Arabidopsis* cDNA and inserted into pAN581 vector via homologous recombination (ABclonal, RM20523). The plasmids used for rice protoplast transformation were extracted using the Promega Wizard Plus Midipreps DNA Purification System. All constructs were transiently expressed in rice protoplasts via PEG-mediated transformation (Yoo et al. 2007). GFP and mCherry fluorescence were detected and imaged using a Leica TCS SP8 laser scanning confocal microscope.

The *35S::OsVPS34-GFP* construct in pCAMBIA1305 background (as described previously) was used to observe the subcellular localization of OsVPS34 in *N. benthamiana* leaves. The full-length CDS of *D53* were merged with mCherry dirved by CaMV 35S promoter by recombined with p1305-mCherry (taking mCherry place of GFP in pCAMBIA1305). The constructions, including *35S::OsVPS34-GFP, 35S::D53-mCherry* and empty pCAMBIA1305 were introduced into *Agrobacterium tumefaciens* strain *EHA105.* The *N. benthamiana* leaves were infected *via Agrobacterium*-mediated transient transformation. The procedure was carried out as previously described (Bao et al. 2023).

### RNA extraction and real-time PCR analysis

Total RNA was extracted from endosperm or other tissue samples using the RNA Prep Pure Plant kit (TIANGEN Biotech, Beijing). Reverse transcription was performed on 1 to 2 µg of total RNA using the HiScript II 1st Strand cDNA Synthesis kit (Takara, 2690A). Real-time PCR was conducted on a Bio-Rad T100 real-time PCR system (Bio-Rad, California, USA) using SYBR Premix Ex Taq mix (Takara, RR420A). The rice *Ubiquitin* (*Ubi*) gene was used as the internal control. Primers used for RT-qPCR are listed in Table S4.

### Y2H assays

OsATG6s, OsVPS38, and OsATG14 were identified in the rice database through alignment with their *Arabidopsis* homologs. *OsATG6* has 3 copies, designated *OsATG6a*, *OsATG6b*, and *OsATG6c*, while *OsVPS38* and *OsATG14* exist as single-copy genes in rice. The full-length CDS of *OsATG6a*, *OsATG6b*, *OsATG6c*, *OsVPS38*, and *OsATG14* were amplified from WT Ningjing 4 and cloned into the pENTRY vector. These were then recombined with pGADT7-GW1 and pGBKT7-GW1 *via* gateway LR reaction (Invitrogen, 2269734) for subsequent sequencing and plasmid extraction. Reagents for the yeast 2-hybrid experiment were purchased from Clontech. Plasmids corresponding to the genes to be tested for interaction were co-transformed into the *Saccharomyces cerevisiae* strain AH109 in various combinations, with appropriate controls. The preparation of competent yeast cells and transformation procedures were carried out according to the *Yeast Two-Hybrid System* handbook (Clontech).

### Firefly LCI assays, BiFC assays, and Co-IP

For firefly luciferase complementation imaging (LCI), the full-length CDSs of *OsVPS34* and *OsATG6b* were initially cloned into the pENTRY vector. These sequences were subsequently inserted upstream of nLUC and cLUC in the pEARLY-nLUC and pEARLY-cLUC vectors (modified by our lab based on pEARLY103) via LR reaction (Invitrogen, 2269734). The resulting constructs were transformed into the *Agrobacterium tumefaciens* strain EHA105 and transiently expressed in *N. benthamiana* leaves (Yan et al. 2024). Luciferase activity was measured 2 to 3 d post-infiltration using the Tanon 5200CE Chemi-Image System as previously described (Chen et al. 2008), and D-Luciferin potassium salt (D8390, 115114-35-9).

For bimolecular fluorescence complementation (BiFC) assays, *OsVPS34* and *OsATG6b* were cloned into the pENTRY vector and recombined with pEARLY-nYNE and pEARLY-YCE (modified by our lab based on pEARLY103), respectively, through LR reaction. The constructed vectors were introduced into *Agrobacterium tumefaciens* strain EHA105 and infiltrated into leaves of *N. benthamiana* leaves. The CDS of *Man1*, *ST*, and *AtVSR2*, which were inserted into p1305-mCherry to merge mCherry, were used for organelle markers (Wang et al. 2010). Observations and imaging were performed on 48 to 72 h post-infiltration using Leica TCS SP8 laser confocal microscope. The CDS of OsATG6b was amplified from pENTRY-OsATG6b and inserted into 1305-3×Flag (modified by our lab based on pCAMBIA1305) vector to make *35S::OsATG6b-3Flag* construction via homologous recombination. *35S::OsATG6b-3Flag* and *35S::OsVPS34-GFP* were co-infiltrated into leaves of *N. benthamiana* leaves. On 2 to 3 d post infiltration, the expression of OsATG6b-3Flag and OsVPS34-GFP were detected by western blotting with anti-FLAG (PM020-7, MBL Life Science) and anti-GFP (11814460001, Roche). The dilution ratio is 1:5,000. Then, we performed Co-IP with anti-GFP beads (D153, MBL Life Science) and immunoblotting with anti-GFP (11814460001, Roche). Anti-IgG (H + L chain) (Mouse) pAb-HRP (MBL, 330) were used as secondary antibodies. All antibodies used in this study were diluted at a ratio of 1:5,000.

### Immunoprecipitation and mass spectrometry

Immunoprecipitation and mass spectrometry (IP-MS) analyses were performed as previously described (Ren et al. 2020). Briefly, 10 g fresh seedlings of 15-d-old *35S::OsVPS34-GFP* T_2_ homozygous were ground in liquid nitrogen and resuspended in 15 mL NB1 buffer (PH = 8.0 50 mM Tris; 0.5 M sucrose; 1 mM 6H_2_O·MgCl_2_; 10 mM EDTA; 5 mM DTT) with freshly added protease inhibitor cocktail (20123ES50, YEASEN). The mixture was incubated at 4 °C for 30 min to extract total protein, followed by centrifugation at 4,000 rpm for 10 min at 4 °C to remove debris. The supernatant was collected for subsequent experiments. To reduce nonspecific binding, 50 µL of prewashed protein G beads with cold NB1 buffer were added to the supernatant and incubated at 4 °C for 1 h at 40 rpm. The mixture was centrifuged 6,000 rpm at 4 °C for 5 min, and the supernatant was retained for immunoprecipitation. Next, 50 µL of prewashed anti-GFP beads (D153, MBL Life Science) with cold NB1 buffer were added to the supernatant and incubated at 4 °C for 2 h. Beads were then collected using a magnetic rack and washed 3 times with cold NB1 buffer with freshly added protease inhibitor cocktail. For elution, 60 µL of NB1 buffer and 15 µL of 5× protein loading buffer (P0015L, Beyotime) were added to the beads, followed by boiling at 95 °C for 5 min. A 5-µL aliquot was subjected to SDS-PAGE, followed by CBB staining and western blotting with anti-GFP (11814460001, Roche) to detect *OsVPS34-GFP* after immunoprecipitation. The remaining sample was separated by SDS-PAGE (10% gel, 120 V, 20 min), and the corresponding gel slice was cut and sent to Beijing Protein Innovation for mass spectrometry analysis. The *35S::GFP* T_2_ homozygous were used as control. All antibodies used in this study were diluted at a ratio of 1:5,000.

### Protoplasts with DEX treatment

The CDS of *SYP61* and *Rha1*, which were inserted into pAN581 to merge mCherry, were used for organelle markers (Wang et al. 2010). The CDS of *Rab5a* and GPA5 were amplified and inserted into pAN580 to merge GFP. The plasmids used for rice protoplast transformation were extracted using the Promega Wizard Plus Midipreps DNA Purification System. All constructs were transiently expressed in rice protoplasts via PEG-mediated transformation. After PEG-mediated transient transformation for 6 to 8 h, rice protoplasts were treated with 50 µM DEX for an additional 8 h before observation (Vermeer et al. 2006). GFP and mCherry fluorescence were detected and imaged using a Leica TCS SP8 laser scanning confocal microscope.

### Measurement of PI3P content in endosperm

The DEX/LY94002 treatment was applied to 3-d-old seedlings for 5 to 7 d, and the leaves were collected for the determination of PI3P content. DMSO treatment was used as the control. About 0.1 g sample of developing endosperm/seedling leaf was ground with liquid nitrogen, and then 250 uL of buffer (50 mM Tris-HCl (pH7.4), 150 mM NaCl, 1 mM EDTA, 1% Triton X-100, protease inhibitor cocktail (20123ES50, YEASEN)) was added. The mixture was extracted on a 4 °C shaker for 30 min, and then centrifuged at 12,000 × *g* for 20 min at 4 °C. The supernatant (containing soluble lipids) was collected, and the precipitate (cell walls and impurities) was discarded. The PI3P content was detected by PI3P detection kit (LM8K957o, SHANG HAI LMAI Bio).

### Subcellular fractionation

The Rab5a-GFP transgenic plants were obtained by Yu Zhang from Nanjing Agricultural University, Nanjing (Zhang et al. unpublished data, 2025). At 3 d after germinating, *Rab5a-GFP* seedlings were treated with 50 µM LY294002 for 10 d, with DMSO as the control. Roots from the seedlings were collected and ground in liquid nitrogen. NB1 buffer (PH = 8.0 50 mM Tris; 0.5 M sucrose; 1 mM 6H_2_O·MgCl_2_; 10 mM EDTA; 5 mM DTT) with freshly added protease inhibitor cocktail (20123ES50, YEASEN) was added into the mortar as the ratio 2 uL/mg. Then the homogenate was transferred to cold 2.0-mL centrifuge tubes for incubation at 4 °C for 1 h. After incubation, the samples were centrifuged at 2,000 × *g* for 5 min, and the cell debris was removed by filtration through coarse cloth. The supernatant was transferred to a new cold 2.0-mL centrifuge tube, with 100 µL taken as the total protein. The remaining supernatant underwent ultracentrifugation at 100,000 × *g* for 120 min at 4 °C to separate the soluble fraction (S100) from the membrane fraction pellet (P100). We added 1/4 volume of 5× loading buffer with soluble fraction, and equal volume 1× loading buffer as total soluble fraction into fraction pellet to resuspend the pellet. Total protein, soluble fraction, and fraction pellet were boiled at 100 °C for 5 min. All samples were analyzed via SDS-PAGE and western blotting. The operation method for the endosperm samples is the same as described above. The following antibodies were used: Anti-OsUGP (Beijing Protein innovation, AbP80276-A-SE, dilution 1:1,000) used as the marker for S100; Anti-Tip3-1 (dilution 1:2,000) used as the marker for P100; Anti-GFP (Roche, 11814460001, dilution 1:5,000); Anti-GPA5 (dilution 1:5,000) was previously described (Ren et al. 2020).

### Statistical analysis

For statistical analysis, significance between 2 samples were determined using Student *t* test (*P < 0.05 and **P < 0.01). PI3P punctate structures were quantified using image J software within a 500-µm^2^ area. The diameters of PBIs and PBⅡs were measured with Leica TCS SP8 software. The scatter plot in the co-localization analysis represents the intensity distribution of all pixels in the 2 channels as a scatter plot, where the higher the degree of co-localization, the more the scatter plot tends to diagonal distribution, and the specific method is as described previously (Zinchuk et al. 2007). PCC analysis statistics were performed as described in previous reports (Zhou et al. 2023).

### Accession numbers

Sequence data for this article can be found in the NCBI database under the following accession numbers: *OsVPS34* (*Os05g0180600*), *GPA5*, (*Os06g0643000*), *Rab5a*/*GPA1* (*Os12g0631100*), *OsATG6a* (*Os01g0681400*), *OsATG6b* (*Os03g0644000*), *OsATG6c* (*Os03g0258500*), *OsVPS15* (*Os02g0796700*), *OsVPS38* (*Os06g0715000*), *OsATG14* (*Os07g0626300*), *D53* (*Os11g0104300*), *Ubiquitin* (*Os03g0234200*), *OsUGP* (*Os09g0553200*), *ARA7* (*At4g19640*), *SYP61* (*At1g28490*), and *Rha1* (*At5g45130*).

## Supplementary Material

kiag154_Supplementary_Data

## Data Availability

The data supporting the findings of this study are available within the article and its Supplementary Data. Additional data are available from the corresponding author upon reasonable request.
