# Supplementary material for: OsVPS34-generated PI3P recruits GPA5/Rab5a to regulate post-Golgi glutelin trafficking in rice endosperm
Source: Plant Physiol. 2026 Mar 18;201(3):kiag154. doi: 10.1093/plphys/kiag154 (PMC13360276; doi:10.1093/plphys/kiag154)
Supplement: kiag154_Supplementary_Data [file kiag154_supplementary_data.zip › Supplementary Figure-Clean.pdf]

Supplementary Figure

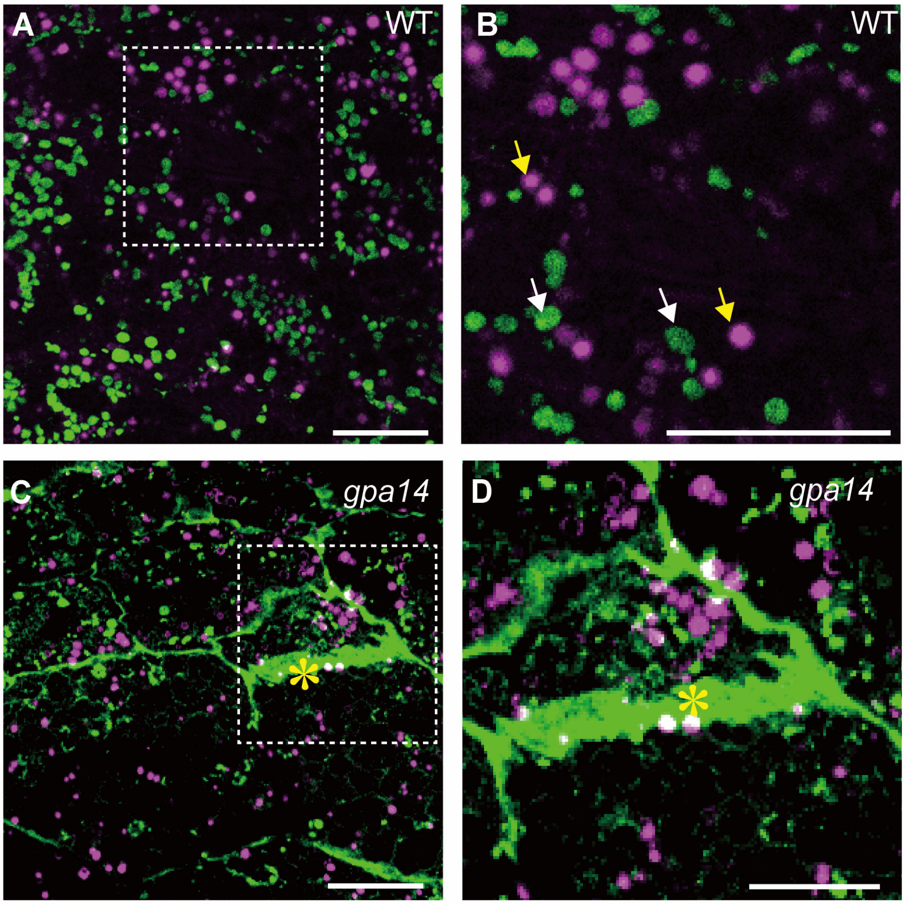

Supplementary Figure S1 Immunofluorescence microscopy was used to observe glutelins and prolamins in developing sub-aleurone cells at 12 DAF in WT and *gpa14* mutant.

(A-D) Secondary antibodies conjugated with Alexa fluor 488 (green) and Alexa fluor 555 (purple) were used to detect antigens recognized by the monoclonal anti-glutelin antibodies (mouse) and polyclonal anti-prolamin antibodies (rabbit), respectively. White arrows indicate PBIIs, yellow arrows indicate PBIs, asterisks mark the PMB structure. Panels B shows magnified views of the white boxed areas in panels A, and panels D shows magnified views of the white boxed areas in panels C, Scale bars = 25 µm in A, C; Scale bars = 10 µm in B, D.

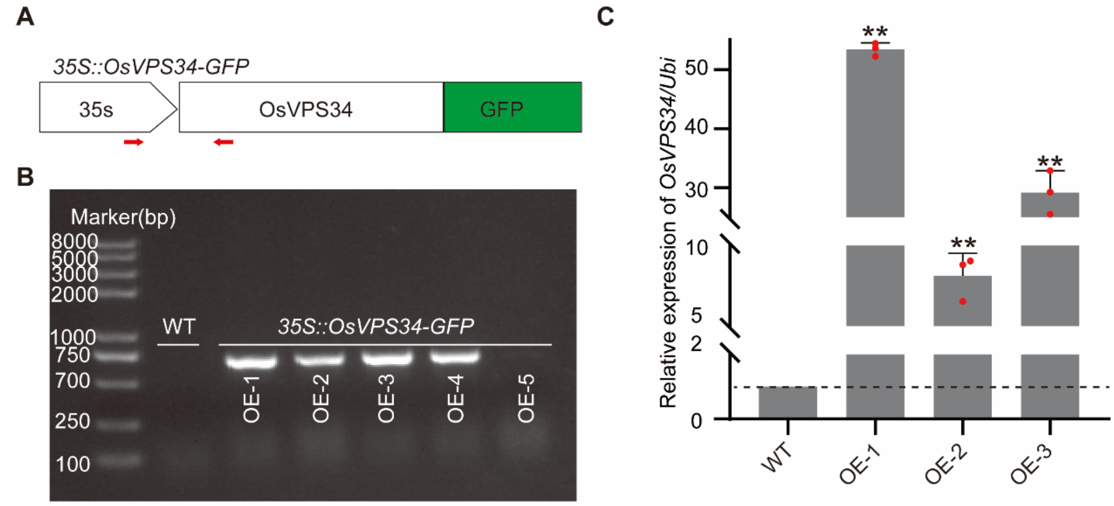

Supplementary Figure S2 Identification of *OsVPS34* transgenic complementary family.

(A) The schematic diagram of the vector structure, with red arrows indicating the locations of the PCR primers. White arrow means *OsVPS34* promoter and white rectangle means the *OsVPS34* CDS. (B) PCR product detection of *35::OsVPS34-GFP* transgenic complementary family. (C) Detection of *OsVPS34* expression level in the 15-day seedling leaves of three overexpression lines. The y-axis represents relative expression levels normalized to the wild type, which is set to 1. Asterisks indicate statistically significant difference between WT and overexpression lines, determined by Student's *t*-tests. Data represent mean ± SD (n = 3; ** $P < 0.01$). *Ubiquitin* (*Ubi*) was used as a control.

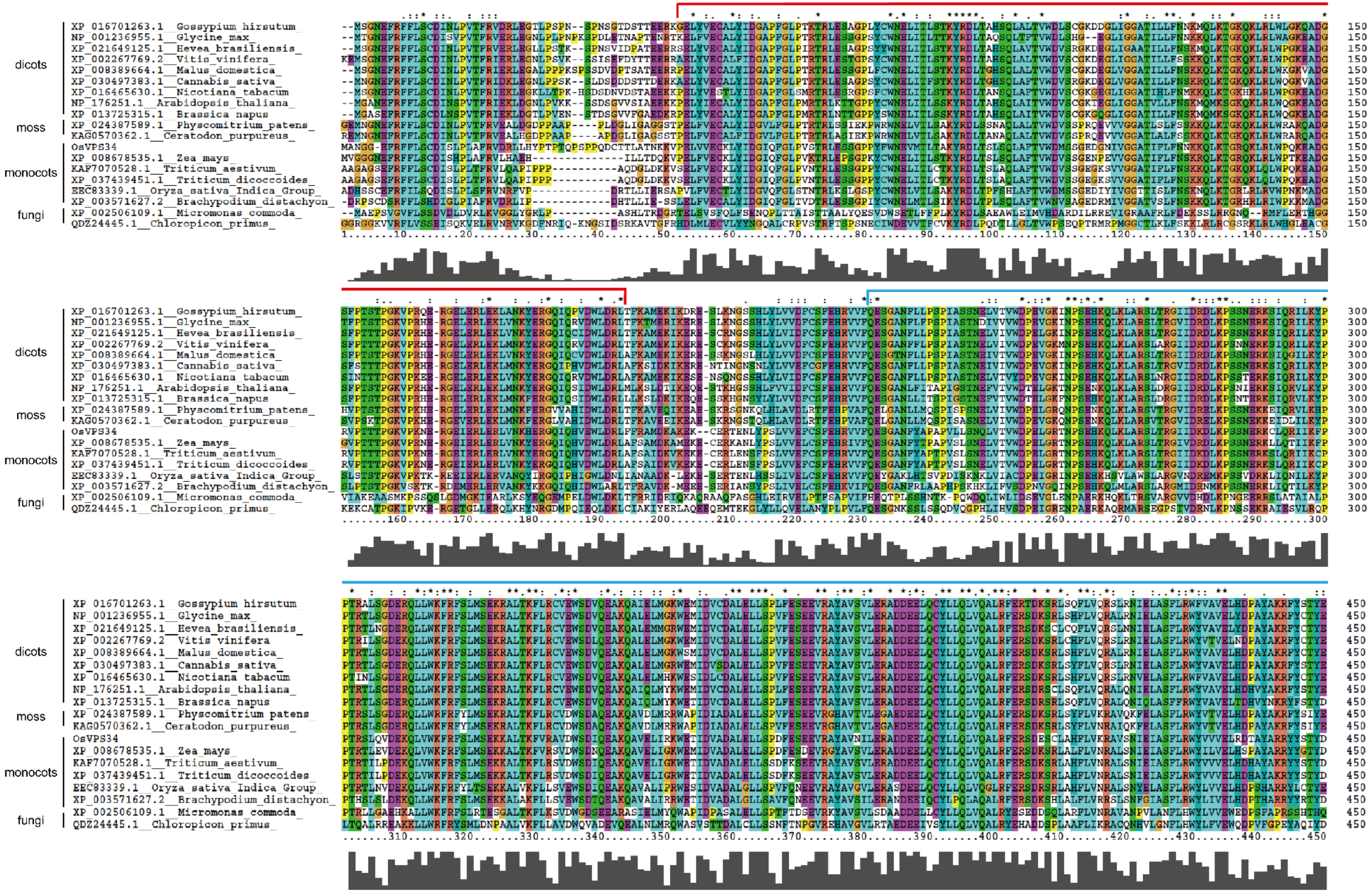
dicots
moss
monocots
fungi
XP_016701263.1 Gossypium hirsutum
NP_001236955.1 Glycine max
XP_021649125.1 Hevea brasiliensis
XP_002267769.2 Vitis vinifera
XP_008389664.1 Malus domestica
XP_030497383.1 Cannabis sativa
XP_016465630.1 Nicotiana tabacum
NP_176251.1 Arabidopsis thaliana
XP_013725315.1 Brassica napus
XP_024387589.1 Physcomitrium patens
KAG0570362.1 Ceratodon purpureus
OsVPS34
XP_008678535.1 Zea mays
KAF7070528.1 Triticum aestivum
XP_037439451.1 Triticum dicoccoides
EEC83339.1 Oryza sativa Indica Group
XP_003571627.2 Brachypodium distachyon
XP_002506109.1 Micromonas commoda
QDZ24445.1 Chloropicon primus

26

27

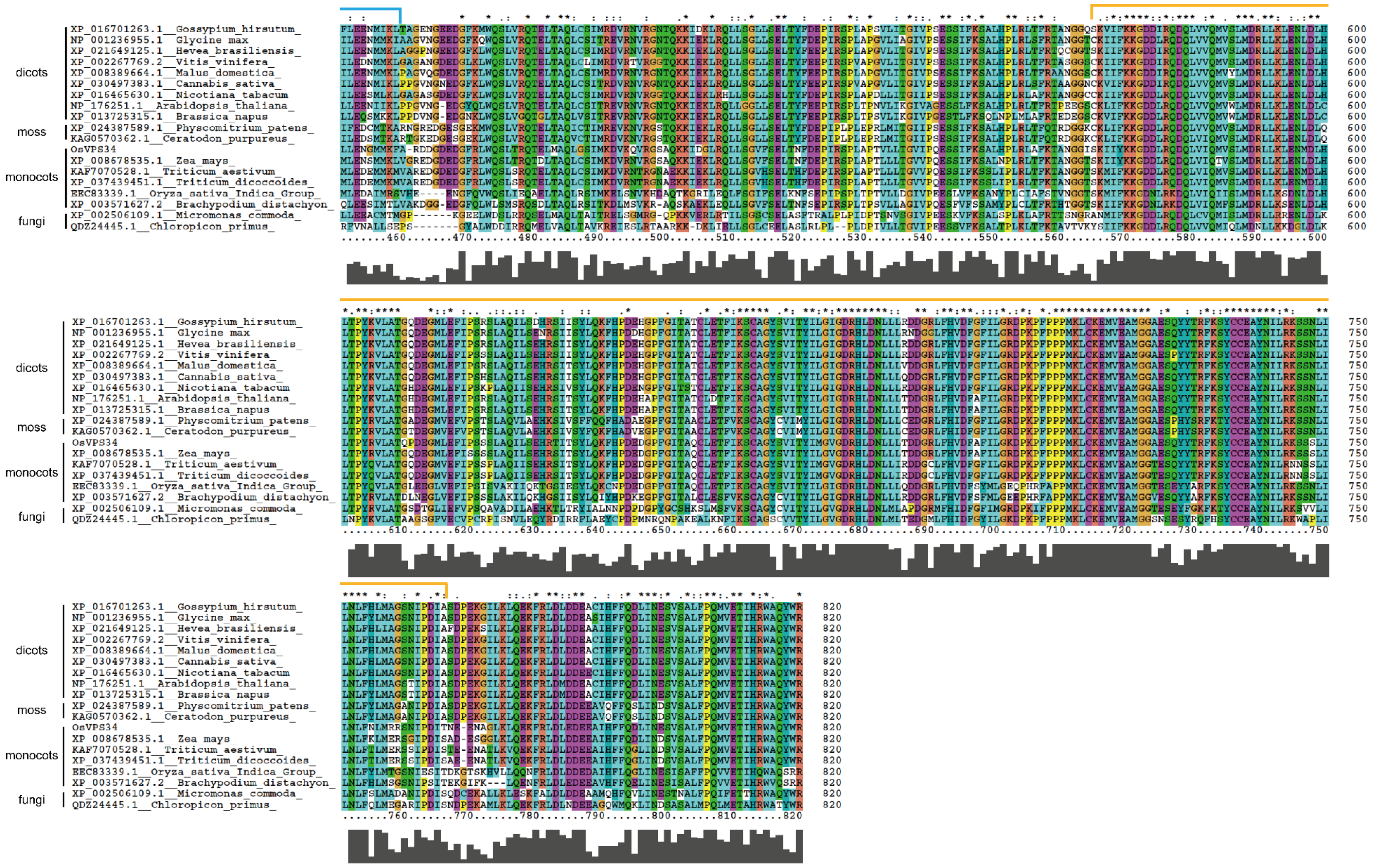
dicots
moss
monocots
fungi
XP_016701263.1__Gossypium_hirsutum_
NP_001236955.1__Glycine_max_
XP_021649125.1__Hevea_brasiliensis_
XP_002267769.2__Vitis_vinifera_
XP_008389664.1__Malus_domestica_
XP_030497383.1__Cannabis_sativa_
XP_016465630.1__Nicotiana_tabacum
NP_176251.1__Arabidopsis_thaliana_
XP_013725315.1__Brassica_napus
XP_024387589.1__Physcomitrium_patens_
KAG0570362.1__Ceratodon_purpureus_
OsVPS34
XP_008678535.1__Zea_mays_
KAF7070528.1__Triticum_aestivum_
XP_037439451.1__Triticum_dicoccoides_
EEC83339.1__Oryza_sativa_Indica_Group_
XP_003571627.2__Brachypodium_distachyon_
XP_002506109.1__Micromonas_commoda_
QDZ24445.1__Chloropicon_primus_
600
750
820

Supplementary Figure S3 Amino Acid Sequence Alignments of OsVPS34 and its homologs in dicots, moss, monocots and fungi using Clustal X2.

The red line represents the PI3K_C2 domain, the blue line represents the PI3ka domain, and the yellow line represents the PI3_PI4_kinase domain. Asterisks and bar chart indicate amino acid conservation.

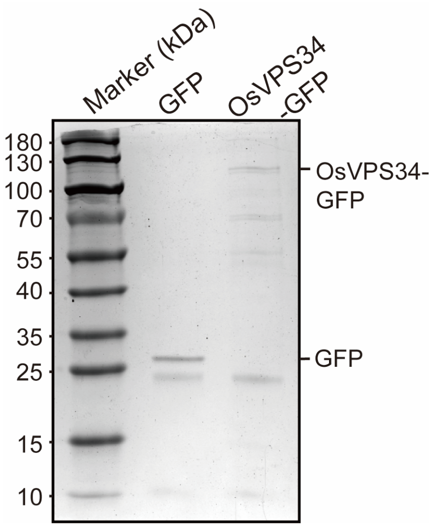

Supplementary Figure S4 SDS-PAGE gel stained by CBB. The 15-day-old rice seedlings from transgenic plants expressing free GFP or OsVPS34-GFP were subjected to immunoprecipitation using anti-GFP magnetic beads. Precipitated proteins with free GFP or OsVPS34-GFP were loaded.

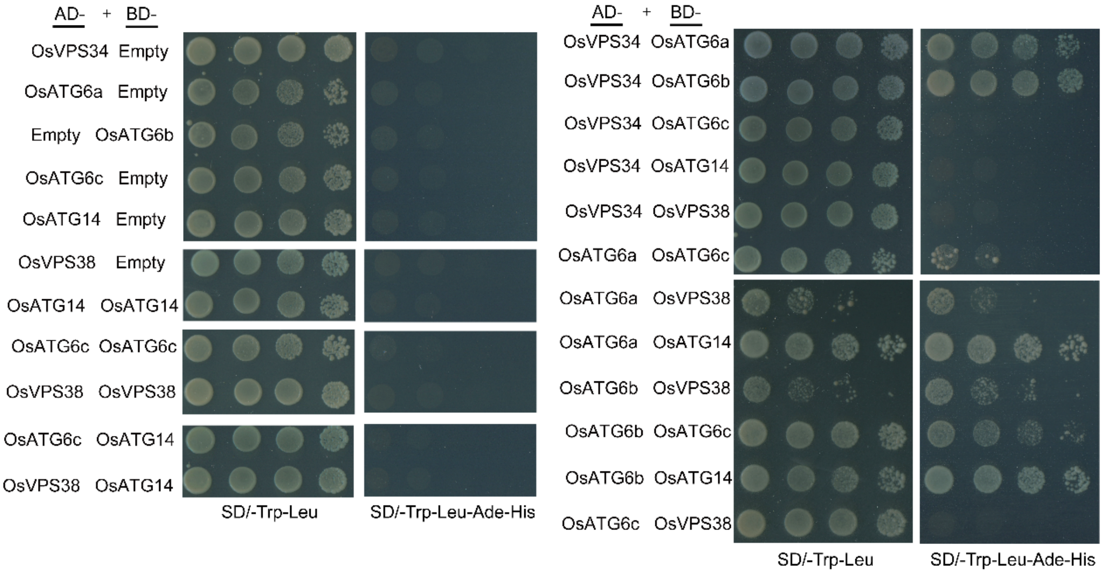

Supplementary Figure S5 The interaction between different subunits of PI3K complex in rice was verified by Y2H.

AD, fused with activation domain; BD, fused with binding domain.

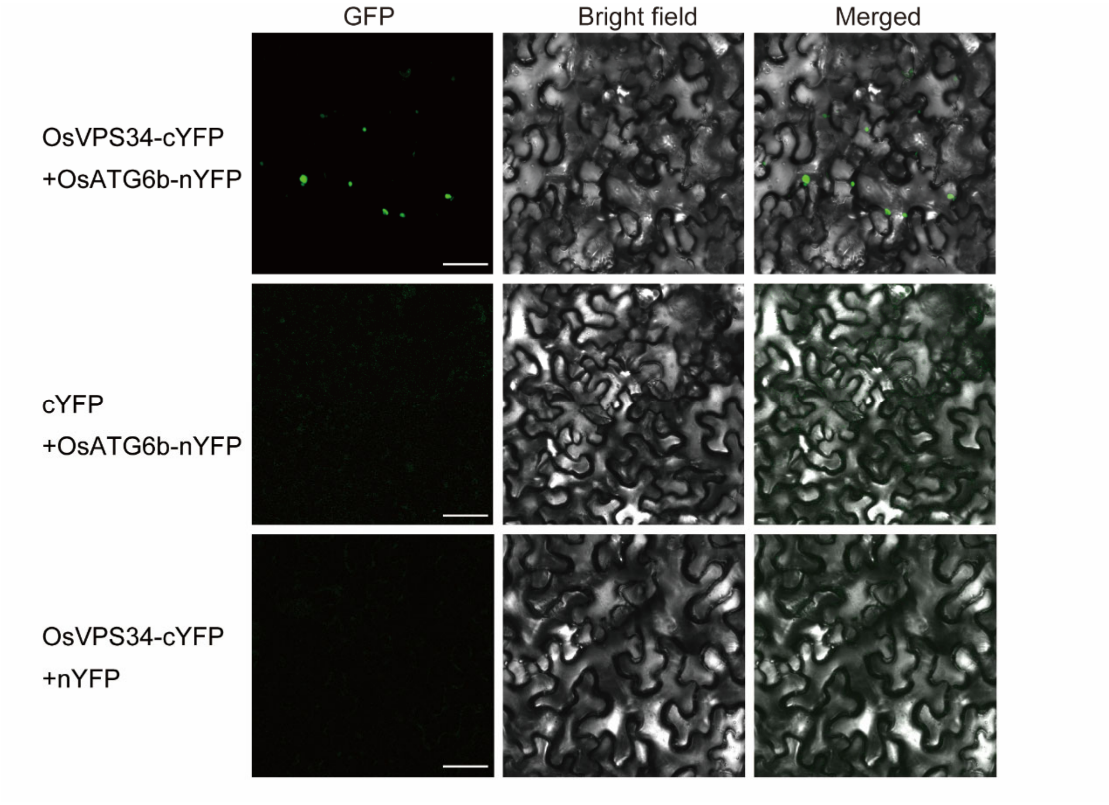

Supplementary Figure S6 BiFC assay shows that OsVPS34 interacts with OsATG6b in *N. benthamiana* leaf cells. Scale bars =50 μm.

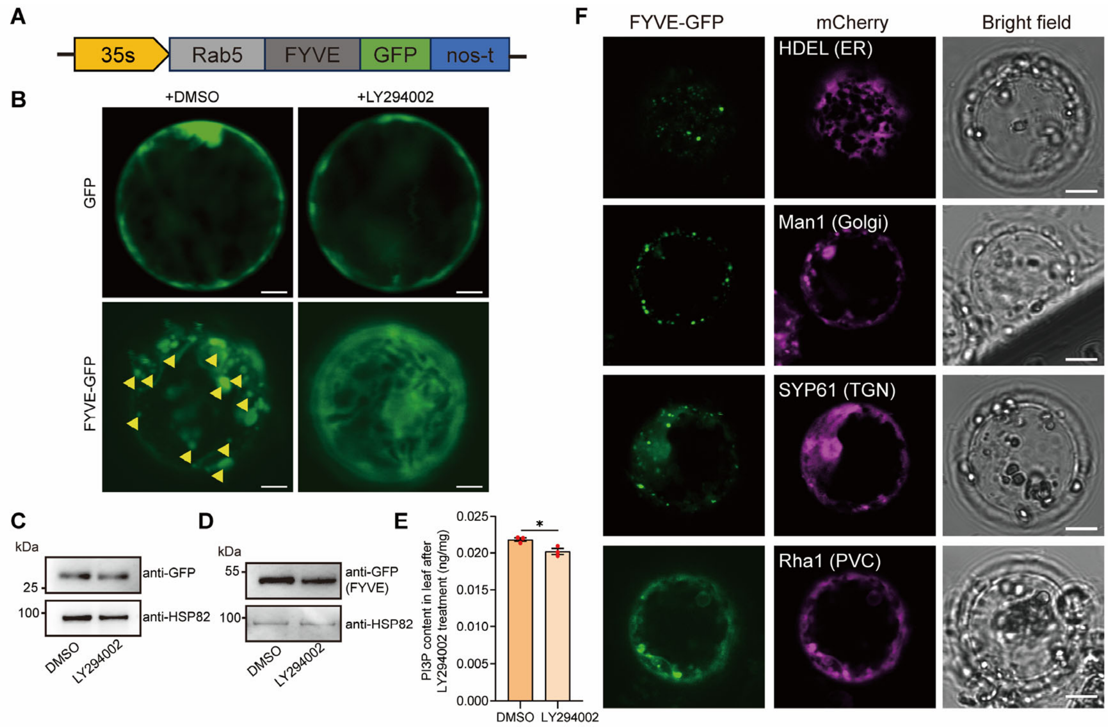

Supplementary Figure S7 Observation of protoplasts treated with LY294002 and single-channel fluorescence observation of GFP and mCherry.

(A) The schematic diagram of the FYVE-GFP vector. The FYVE domain, contain the binding domain Rab5, and the FYVE domain, which originate from the C-terminal region of EEA1 (amino acids 1257 to 1411). (B) Observation of PI3P localization after 50 µM LY294002 treatment of protoplasts. Scale bars = 10 µm. (C, D) Detection of the level of GFP in total proteins after LY294002 treatment of protoplasts. (E) Detection of PI3P content in the leaves of treated seedlings with LY294002. Asterisks indicate the statistical significance between the DMSO and LY294002 treatment, determined by Student's *t*-tests. Data represent mean ± SD ($n = 3$; $^{*}P < 0.05$).

(F) FYVE-GFP was transiently expressed in rice protoplasts alongside various organelle markers: HDEL-mCherry (ER marker), Man1-mCherry (Golgi marker), mCherry-SYP61 (TGN marker), and mCherry-Rha1 (PVC marker). The samples shown in this figure are the same as those shown in Figure 7F. Scale bars = 10 µm.

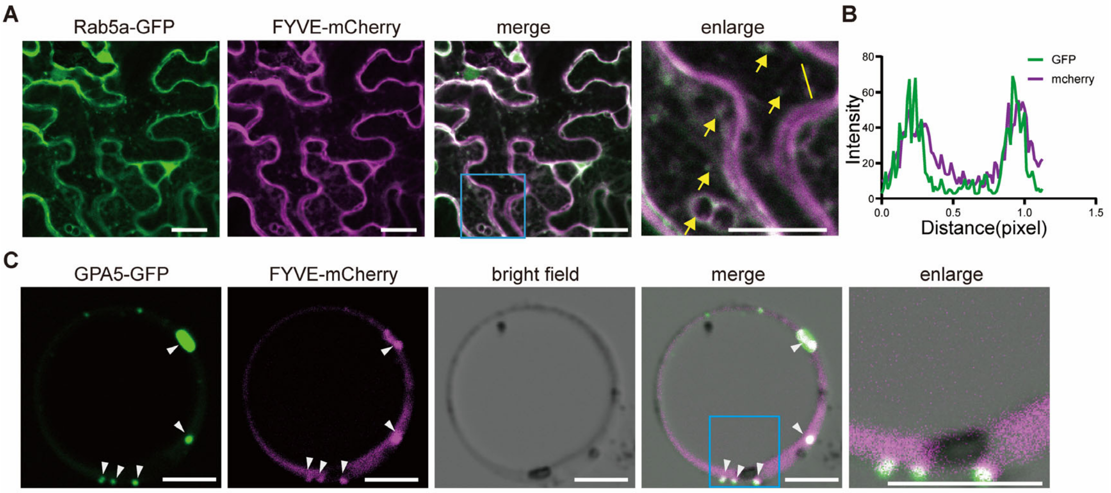

Supplementary Figure S8 Localization analysis of Rab5a and GPA5 with PI3P.

(A) Rab5a-GFP and FYVE-mCherry were co-expressed in *N.benthamiana* leaf cells and observed by laser confocal. The yellow arrow indicates the colocalization sites of FYVE and Rab5a. Scale bars = 25 μm; enlarge bar = 25 μm. (B) A graph with pixel intensity along yellow line. (C) GPA5-GFP and FYVE-mCherry were co-expressed in rice protoplasts. The white arrow indicates the colocalization sites of FYVE and GPA5. Scale bars = 10 μm; enlarge bar = 10 μm.

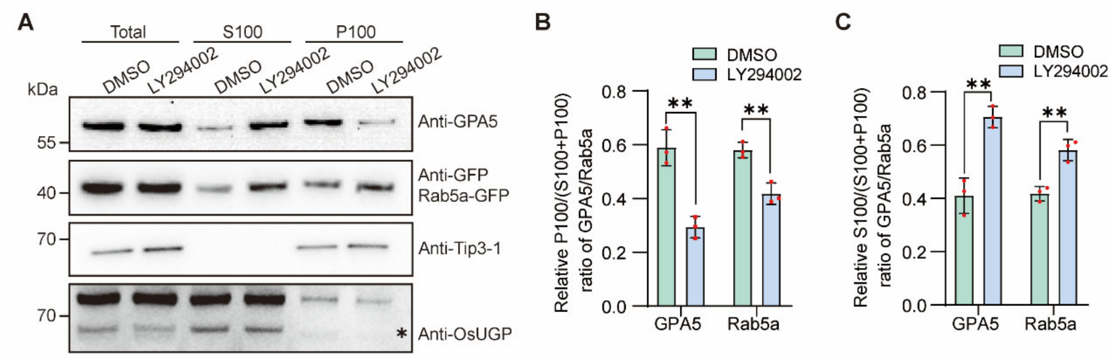

Supplementary Figure S9 Effect of LY294002 on the subcellular distribution of GPA5 and Rab5a.

(A) Immunoblot analysis of GPA5 and Rab5a-GFP in the membrane (P100) and cytoplasmic (S100) fractions after LY294002 treatment. Rice seedlings were treated with LY294002 (50 μM) for 4 days post-germination. Total protein was extracted from young roots and subjected to ultracentrifugation (100,000 g for 1 h) to separate P100 (membrane fraction) and S100 (cytoplasmic fraction). Anti-Tip3-1 was used as a P100 marker, and anti-OsUGP as a S100 marker. Asterisks indicate OsUGP bands.

(B-C) Quantification of Rab5a-GFP and GPA5 distribution in P100 and S100 fractions based on immunoblot band intensity from (A). Band intensities were quantified using

ImageJ software. Asterisks indicate statistically significant difference between WT and overexpression lines, determined by Student's *t*-tests. Data represent mean ± SD (n = 3; ** $P < 0.01$).

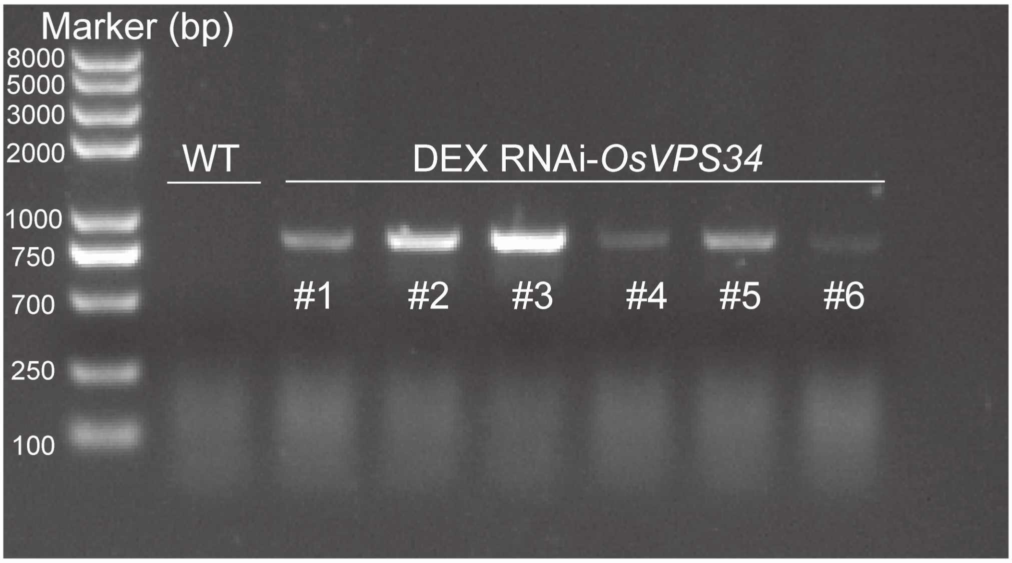

Supplementary Figure S10 The gel image of DEX RNAi-*OsVPS34* transgenic plants. # 1~6 indicates different transgenic plants.

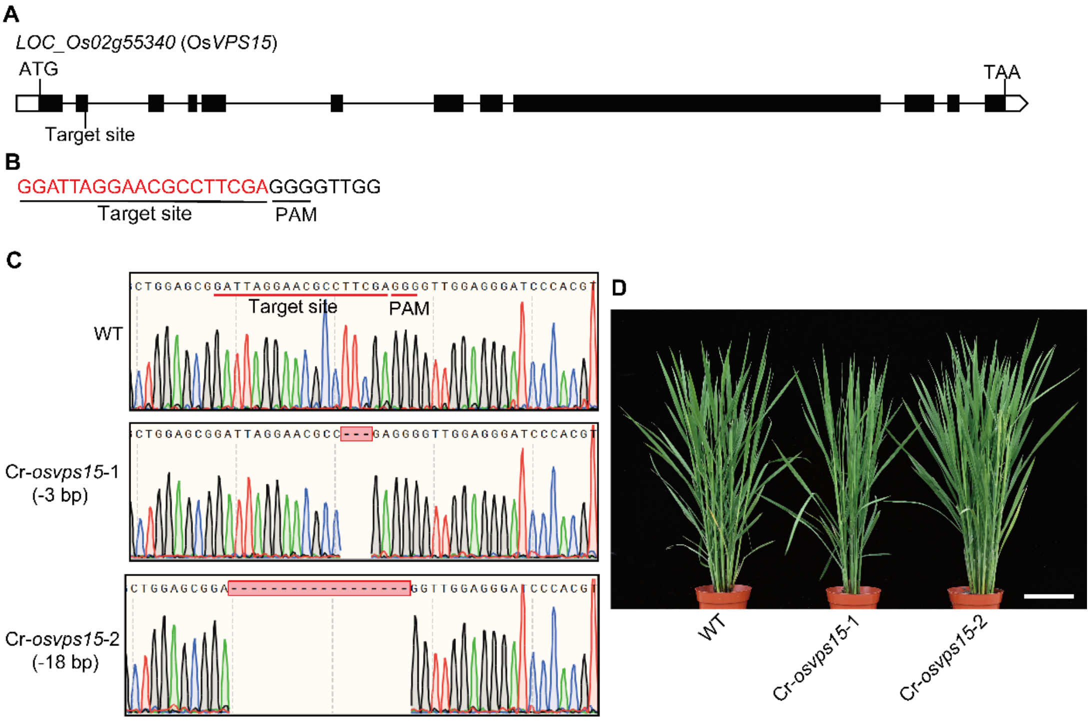

Supplementary Figure S11 Analysis of *LOC_Os02g55340* (*OsVPS15*) knockout lines

generated by CRISPR/Cas9.

(A) Diagram of target sites in the second exon of *LOC_Os02g55340*. White boxes represent UTR, Black boxes represent exons, lines represent introns, ATG and TAA indicate the start and stop codons, respectively. (B)The sgRNA target sequence of *LOC_Os02g55340.* PAM, protospacer-adjacent motif. (C) DNA sequencing results showing the deletion in *LOC_Os02g55340* obtained by CRISPR/Cas9. Cr-*osvps15*-1 and Cr-*osvps15*-2 are homozygous mutants with a 3 bp and 18 bp deletions at target site. (D) Plant phenotypes of WT and knockout line. Scale bar = 10 cm.

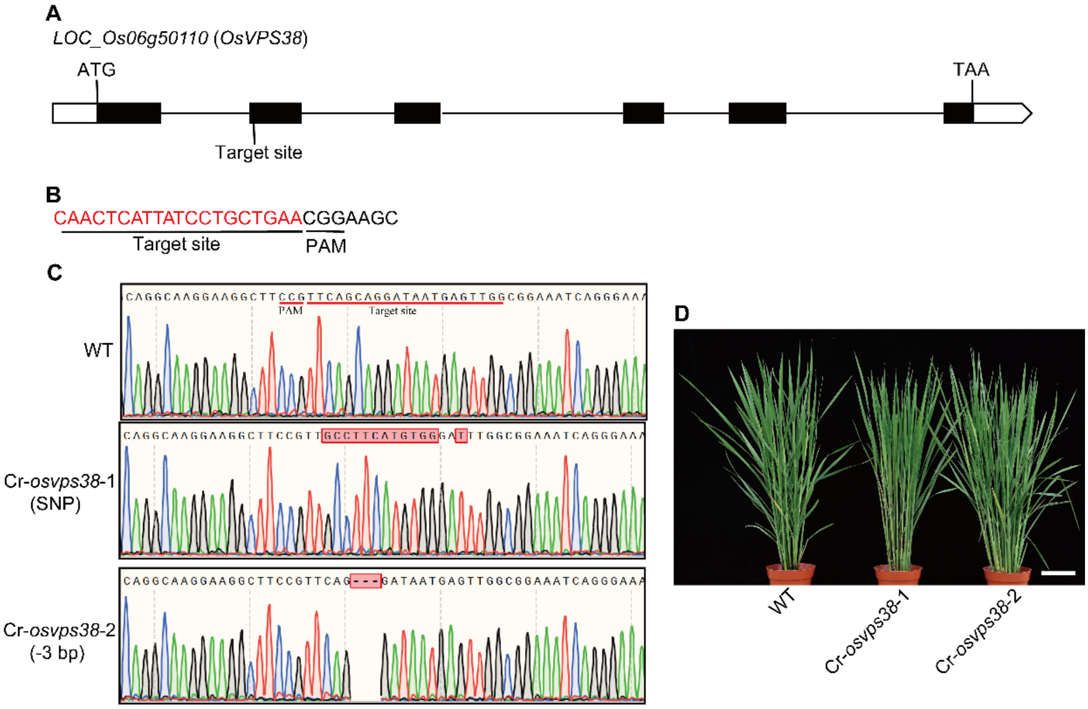

Supplementary Figure S12 Analysis of *LOC_Os06g50110* (*OsVPS38*) knockout lines generated by CRISPR/Cas9.

(A) Diagram of target sites in the second exon of *LOC_Os06g50110*. White boxes represent UTR, Black boxes represent exons, lines represent introns, ATG and TAA indicate the start and stop codons, respectively. (B)The sgRNA target sequence of *LOC_Os06g50110.* PAM, protospacer-adjacent motif. (C) DNA sequencing results showing the deletion in *LOC_Os06g50110* obtained by CRISPR/Cas9. Cr-*osvps38*-1 and Cr-*osvps38*-2 are homozygous mutants with a 13 bp SNP and 3 bp deletions at target site. (D) Plant phenotypes of WT and knockout line. Scale bar = 10 cm.

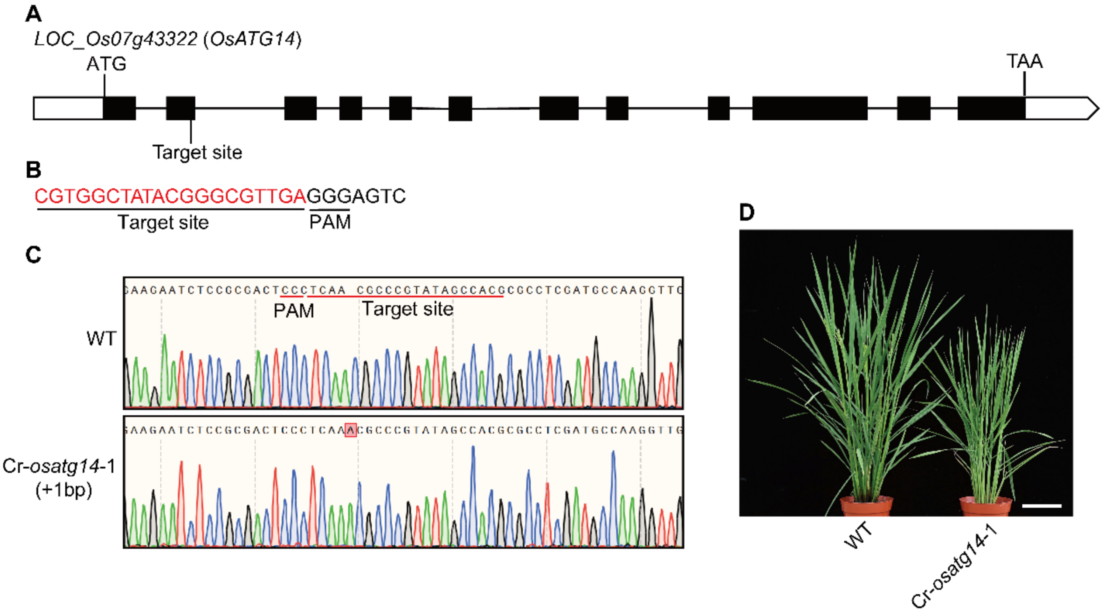

Supplementary Figure S13 Analysis of *LOC_Os07g43322* (*OsATG14*) knockout lines generated by CRISPR/Cas9.

(A) Diagram of target sites in the second exon of *LOC_Os07g43322*. White boxes represent UTR, Black boxes represent exons, lines represent introns, ATG and TAA indicate the start and stop codons, respectively. (B)The sgRNA target sequence of *LOC_Os07g43322*. PAM, protospacer-adjacent motif. (C) DNA sequencing results showing the deletion in *LOC_Os07g43322* obtained by CRISPR/Cas9. Cr-*osatg14*-1 is homozygous mutants with 1 bp insertion at target site. (D) Plant phenotypes of WT and knockout line. Scale bar = 10 cm.
